# Atom–Cluster Synergy in Scalable Fe–Ru Dual‐Site Architectures Accelerates Alkaline Hydrogen Evolution

**DOI:** 10.1002/adma.74980

**Published:** 2026-09-10

**Authors:** Jae‐Hoon Baek, Seong Hyeon Kweon, Sun Gwan Cha, Se Jung Lee, Jinwoo Baek, Dong Hyeok Kwon, Hojeong Lee, Youngkook Kwon, Sang Kyu Kwak, Jong‐Beom Baek

**Affiliations:** ^1^ Department of Energy and Chemical Engineering/Center For Dimension‐Controllable Organic Frameworks Ulsan National Institute of Science and Technology (UNIST) Ulsan South Korea; ^2^ Department of Energy and Chemical Engineering Ulsan National Institute of Science and Technology (UNIST) Ulsan South Korea; ^3^ Graduate School of Carbon Neutrality Ulsan National Institute of Science and Technology (UNIST) Ulsan South Korea; ^4^ Department of Materials Science and Engineering Pohang University of Science and Technology (POSTECH) Pohang South Korea; ^5^ Department of Chemical and Biological Engineering Korea University Seoul South Korea; ^6^ UNIST InnoCORE Hydro*studio Ulsan National Institute of Science and Technology (UNIST) Ulsan South Korea

**Keywords:** alkaline hydrogen evolution, anion exchange membrane water electrolyzer (AEMWE), dual‐site electrocatalyst, mechanochemical synthesis, single‐atom catalyst

## Abstract

Sluggish initial water dissociation (the Volmer step) severely limits alkaline water electrolysis. We report a highly scalable mechanochemical strategy to construct a dual‐site electrocatalyst, Ru_NC_@Fe_1_NC, that spatially decouples water activation and hydrogen recombination. Harnessing the high‐energy impact of iron media and graphite, a solvent‐free mechanochemical process generates a defective carbon matrix anchoring isolated, oxophilic Fe single atoms (Fe_1_), followed by the targeted deposition of ruthenium nanoclusters (Ru_NC_). This atomic‐level division of catalytic labor fundamentally accelerates the alkaline hydrogen evolution reaction. Ru_NC_@Fe_1_NC requires an overpotential of only 13.8 mV at 10 mA cm^−2^ with an ultralow Ru loading (∼2 wt%). In a practical anion exchange membrane water electrolyzer (AEMWE), this catalyst drives a current density of 1.0 A cm^−2^ at merely 1.66 V and sustains over 1100 h of continuous operation with negligible decay. Mechanistic studies—combining isotopic substitution, in situ Raman spectroscopy, time‐resolved hydrogen accumulation–stripping analysis, and density functional theory—reveal that the Fe_1_ sites readily adsorb water and selectively lower the O–H cleavage barrier, rapidly feeding hydrogen intermediates to the adjacent Ru_NC_ for efficient hydrogen desorption. This work establishes a robust blueprint for designing synergistic dual‐site architectures to circumvent kinetic bottlenecks in green hydrogen production.

## Graphical abstract

A mechanochemically synthesized dual‐site electrocatalyst (Ru_NC_@Fe_1_NC) accelerates alkaline hydrogen evolution via atomic‐level spatial decoupling. Oxophilic Fe single atoms effectively dissociate water, while adjacent Ru nanoclusters rapidly recombine hydrogen. Produced at a 10‐gram scale, this architecture delivers exceptional industrial‐level performance in practical anion exchange membrane water electrolyzers, sustaining 1.0 A cm^−2^ at 1.66 V for over 1100 h.

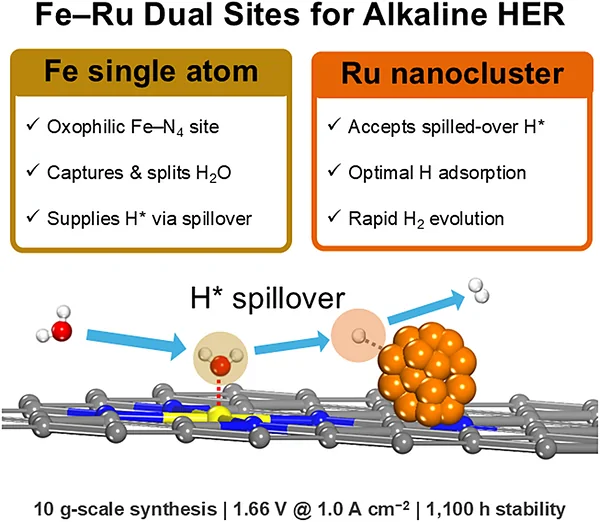

## 1 Introduction

The accelerating global transition toward carbon neutrality has positioned hydrogen as a pivotal energy carrier for decarbonizing hard‐to‐abate sectors and mitigating the intermittency of renewable energy systems. Among the various hydrogen production technologies, water electrolysis powered by renewable electricity is widely regarded as the most viable pathway to truly green hydrogen [1]. Consequently, the development of efficient, durable, and scalable electrolyzer technologies has become a central challenge at the intersection of materials science, electrochemistry, and energy engineering [2, 3]. The field of commercial water electrolysis is currently dominated by alkaline water electrolysis (AWE) and proton exchange membrane water electrolysis (PEMWE) [4]. AWE benefits from mature manufacturing processes and earth‐abundant materials yet suffers from intrinsically low current densities and sluggish reaction kinetics. In contrast, PEMWE enables high current density operation and rapid dynamic response; however, its heavy dependence on scarce noble metals and expensive system components severely limits large‐scale deployment [5]. Anion exchange membrane water electrolysis (AEMWE) has therefore emerged as an attractive alternative, combining the advantages of both systems, while its commercialization remains hindered by insufficient electrocatalyst activity and durability under industrially relevant conditions [6].

From a fundamental perspective, the hydrogen evolution reaction (HER) in alkaline media is intrinsically slower than in acidic environments [7]. Unlike acidic HER, which proceeds via direct proton reduction, alkaline HER requires an additional water dissociation step to generate reactive hydrogen intermediates. This Volmer step, governed by water adsorption and O─H bond cleavage, constitutes the primary kinetic bottleneck in alkaline electrolysis. Consequently, the rational design of electrocatalysts that simultaneously promote water activation and maintain favorable hydrogen adsorption energetics is essential for advancing AEMWE technologies [6, 8]. Ruthenium (Ru)‐based catalysts have attracted increasing attention as promising alternatives to platinum (Pt) for alkaline HER, exhibiting comparable or even superior intrinsic activity at a substantially lower cost [9, 10]. However, pristine Ru surfaces still suffer from sluggish water dissociation kinetics, indicating that further structural and electronic modulation is required to fully exploit their catalytic potential under alkaline conditions [11].

Recent studies have demonstrated that oxophilic single‐atom sites can play a decisive role in overcoming the intrinsic limitations of alkaline HER by directly facilitating water adsorption and dissociation [12, 13, 14]. These isolated atomic centers function not merely as electronic modifiers but as genuine catalytic sites that accelerate the Volmer step and reconfigure the reaction pathway. For example, oxophilic Ce single atoms induce an active‐site reversal mechanism, wherein water dissociation preferentially occurs at Ce–N coordination centers, followed by hydrogen spillover to adjacent metal sites for H_2_ evolution [13]. Similarly, Ga single atoms integrated with Ru‐based catalysts enable an efficient dual‐site architecture, delivering outstanding alkaline HER performance in practical AEMWE devices [14]. Collectively, these studies highlight spatially decoupled water activation and hydrogen recombination as a powerful strategy for accelerating alkaline HER kinetics [15].

Among these oxophilic elements, iron (Fe) stands out as one of the most abundant transition metals, offering an unparalleled combination of availability and cost‐effectiveness for scalable catalyst manufacturing. Computational screenings across the M–N_4_ series have established that the affinity of single‐atom sites toward oxygen‐containing species varies systematically with the metal center, and Fe–N_4_ has been identified as a representative site whose oxygen affinity lies within the catalytically effective range [16]. Fe single atoms have been well established as benchmark active sites in oxygen reduction reaction (ORR) catalysis, owing to their pronounced oxophilicity and highly tunable electronic structure [17, 18, 19]. In particular, Fe–N_x_ coordination environments are well known to strongly stabilize oxygenated intermediates and reduce the activation barriers associated with O─O bond cleavage. Despite these attributes, the role of Fe single atoms in the alkaline HER has remained largely unexplored, even though their intrinsic affinity toward oxygen‐containing species could be highly advantageous for promoting water adsorption and O─H bond dissociation during the Volmer step. This disconnect reveals an unexploited opportunity to repurpose Fe single‐atom chemistry—originally optimized for the ORR—toward the alkaline HER through its rational integration with hydrogen‐affinitive metal sites. Nevertheless, critical challenges remain. Most reported oxophilic single‐atom systems rely on complex synthesis procedures, face limited scalability, and require relatively high noble metal loadings, collectively hindering their translation to practical AEMWE applications. Thus, the development of a scalable, cost‐effective catalyst architecture that synergistically integrates oxophilic single‐atom sites with hydrogen‐affinitive metal domains under realistic operating conditions remains a pressing challenge.

In this study, we present a highly efficient and scalable alkaline HER electrocatalyst featuring the synergistic coexistence of oxophilic Fe single atoms (Fe_1_) and Ru nanoclusters (Ru_NC_) co‐anchored on nitrogenated carbon (NC), denoted as Ru_NC_@Fe_1_NC. This architecture is constructed by initially synthesizing the highly defective Fe_1_NC host via a solvent‐free mechanochemical ball‐milling strategy, followed by the targeted deposition of Ru. This approach enables facile production at the 10‐gram scale with an ultralow Ru loading of ∼2 wt%, demonstrating robust practical manufacturability. Aberration‐corrected scanning transmission electron microscopy (STEM), Mössbauer spectroscopy, and x‐ray absorption fine structure (XAFS) analyses definitively confirm the atomic dispersion of the Fe sites and their intimate integration with the Ru nanoclusters. Mechanistic investigations—combining kinetic isotope effect (KIE) experiments, CO stripping, in situ Raman spectroscopy, time‐resolved hydrogen accumulation–stripping analysis, and density functional theory (DFT) calculations—unveil a distinct functional segregation of the active sites. The highly oxophilic Fe_1_ centers preferentially adsorb and dissociate water to fundamentally lower the kinetic barrier of the Volmer step, while the adjacent Ru nanoclusters provide optimal sites for rapid H–H recombination. This spatial decoupling of water activation and hydrogen evolution effectively mitigates the intrinsic kinetic bottlenecks of alkaline environments. Consequently, Ru_NC_@Fe_1_NC delivers high intrinsic activity, requiring an overpotential of only 13.8 mV to reach 10 mA cm^−2—^less than half that of commercial Pt/C (29.4 mV). Furthermore, when implemented in a practical AEMWE single‐cell device, Ru_NC_@Fe_1_NC achieves an industrial‐level current density of 1.0 A cm^−2^ at 1.66 V and sustains stable operation for 1100 h without noticeable degradation. Ultimately, this work establishes a scalable design paradigm for robust alkaline HER catalysts, providing fundamental insights into how oxophilic single atoms and metallic nanoclusters can be cooperatively engineered to circumvent intrinsic kinetic barriers.

## 2 Results and Discussion

### 2.1 Design and Structural Characterization

We designed a top‐down mechanochemical strategy utilizing ball‐milling‐driven abrasion for the solvent‐ and precursor‐free, gram‐scale synthesis of single‐atom catalysts (Figure 1) [20]. Iron (Fe) single atoms stabilized on a nitrogenated carbon support (Fe_1_NC) were first synthesized via a one‐step ball‐milling process. Specifically, graphite and nitrogen gas (N_2_) were loaded into a milling container alongside iron balls and subjected to high‐energy mechanical impact. In this configuration, the iron balls act simultaneously as the milling medium and the intrinsic iron source; the high‐energy impact drives the atomization of Fe species from the ball surface while generating abundant defects within the graphitic framework. Concurrently, molecular nitrogen is activated and doped into the defective carbon lattice, capturing the atomized Fe species to form atomically dispersed Fe–N coordination sites. Utilizing the as‐prepared Fe_1_NC as a host matrix, Ru nanoclusters (Ru_NC_) were subsequently deposited via solution‐assisted impregnation followed by thermal reduction to construct the Ru_NC_ on Fe_1_NC (Ru_NC_@Fe_1_NC) catalyst. Briefly, the Fe_1_NC support was uniformly dispersed in an *N*‐methyl‐2‐pyrrolidone (NMP) solution containing Ru (III) chloride hydrate (RuCl_3_·xH_2_O) to ensure intimate interfacial contact. During the subsequent thermal reduction under a hydrogen atmosphere, strong Fe–N coordination preserves the isolated Fe single atoms (Fe_1_), whereas the Ru species are selectively reduced to nucleate into Ru nanoclusters on the nitrogenated carbon (NC) support. This divergent structural evolution establishes a well‐defined, heterogeneous dual‐site architecture featuring the coexistence of oxophilic Fe_1_ sites and hydrogen‐affinitive Ru nanoclusters. Highlighting the practical viability of this approach, the robust scalability of the entire synthesis was experimentally validated by the successful single‐batch preparation of ∼10 g of the final Ru_NC_@Fe_1_NC catalyst (Figure S1).

**FIGURE 1:**
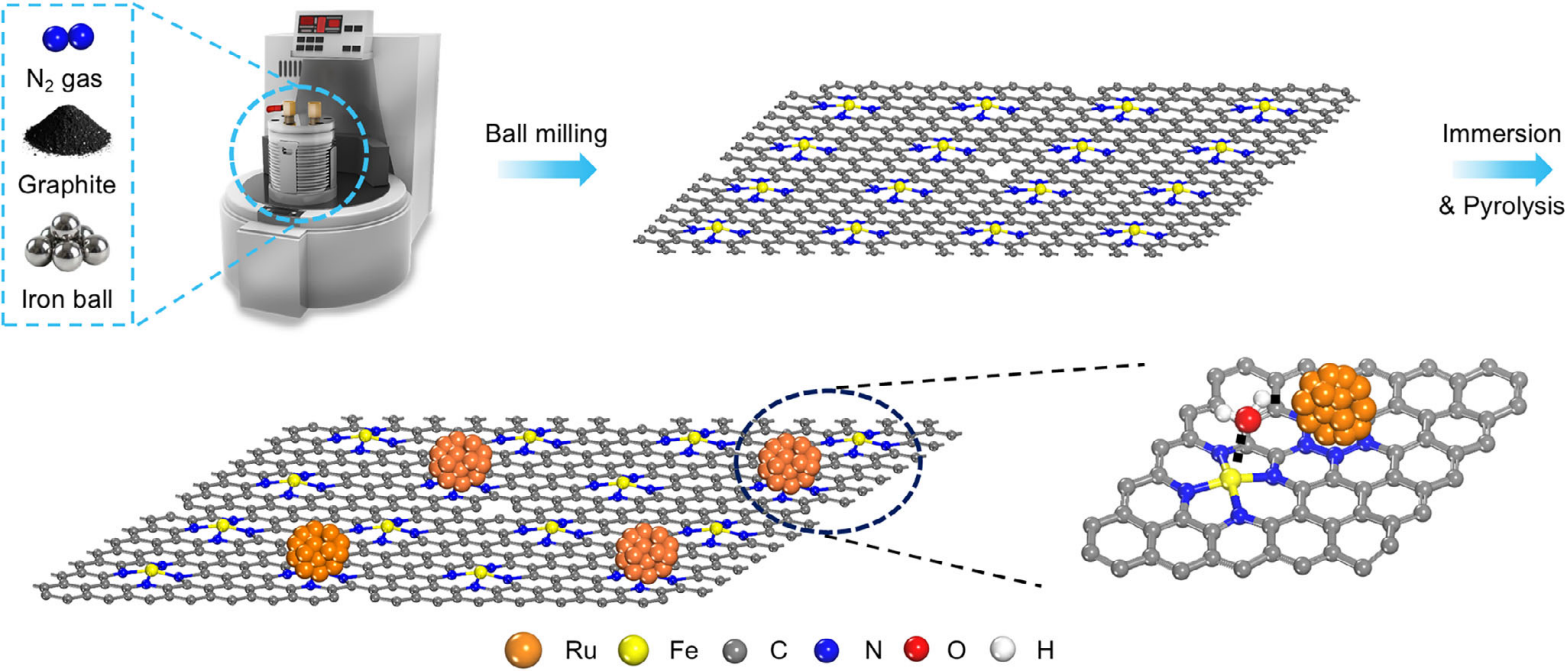
Schematic illustration of the top‐down mechanochemical synthesis and structural evolution of the heterogeneous dual‐site Ru_NC_@Fe_1_NC catalyst, highlighting the solvent‐free fabrication of the defective Fe_1_NC matrix and the subsequent targeted deposition of Ru nanoclusters.

To unambiguously elucidate the specific catalytic role of the Fe single atoms, a Fe‐free control catalyst was deliberately prepared by suppressing Fe atomization during the ball‐milling step. By reducing the rotational speed, Fe abrasion from the milling media was effectively inhibited, yielding a pristine nitrogenated carbon support (NC). Notably, the reduced rotational speed was systematically determined by screening the milling conditions, such that the nitrogen content of the NC support (7.05 wt%) closely matches that of Fe_1_NC (7.47 wt%) while no Fe incorporation occurs, ensuring that the two supports offer a comparable nitrogenated carbon environment (Table S1). Following identical Ru deposition procedures on this Fe‐free support, the Ru species evolved into a mixed population of Ru single atoms (Ru_1_) and nanoclusters (Ru_NC_), yielding the reference catalyst denoted as Ru_NC_@Ru_1_NC (Figure S2). Finally, inductively coupled plasma optical emission spectroscopy (ICP‐OES) confirmed that the Ru loadings were comparable between Ru_NC_@Fe_1_NC and Ru_NC_@Ru_1_NC, with Fe exclusively detected in the Ru_NC_@Fe_1_NC catalyst (Table S2).

The spatial arrangement of the dual‐site configuration was directly examined using high‐angle annular dark‐field scanning transmission electron microscopy (HAADF‐STEM). In both the Ru_NC_@Fe_1_NC and Ru_NC_@Ru_1_NC samples, Ru_NC_ of similar dimensions were uniformly anchored across the NC support. Importantly, individual Fe_1_ sites were clearly visible in Ru_NC_@Fe_1_NC, whereas the Fe‐free Ru_NC_@Ru_1_NC reference primarily contained isolated Ru_1_ sites, demonstrating the effective generation of single‐atom metal centers (Figures 2a and S3). Furthermore, energy‐dispersive x‐ray spectroscopy (EDS) mapping confirmed the uniform elemental distribution of Ru and Fe within the Ru_NC_@Fe_1_NC architecture, attesting to its structural and compositional homogeneity (Figures S4 and S5).

^57^Fe Mössbauer spectroscopy was utilized to precisely determine the chemical state of Fe. Following high‐temperature annealing, the Fe_3_C‐related sextet signal observed in the as‐milled sample was eliminated (Figures 2b and S6) [20]. This result verified that the thermal process drove the full conversion of metastable Fe clusters into stable, atomically dispersed Fe_1_ sites embedded in the carbon host. In agreement with these results, high‐power x‐ray diffraction (HP‐XRD) showed no crystalline metallic phases in either Ru_NC_@Fe_1_NC or Ru_NC_@Ru_1_NC, displaying only a broad diffraction peak at approximately 25.4° attributed to the (002) plane of turbostratic carbon (Figure S7). The absence of long‐range crystallographic ordering provided additional evidence that the metal components existed exclusively as single atoms and ultrasmall nanoclusters. The surface chemical states of the catalysts were subsequently probed by x‐ray photoelectron spectroscopy (XPS) (Figures S8 and S9). In both Ru_NC_@Fe_1_NC and Ru_NC_@Ru_1_NC, the N 1s spectra were deconvoluted into pyridinic N, metal‐coordinated N (Fe–N and Ru–N, respectively), graphitic N, and oxidized N species, indicating that the nitrogenated carbon framework was well preserved after Ru deposition. Meanwhile, the Ru 3p spectra of Ru_NC_@Fe_1_NC were dominated by the metallic Ru° component, consistent with its cluster‐form Ru speciation, whereas Ru_NC_@Ru_1_NC exhibited a markedly enhanced Ru^n+^ contribution, reflecting the coexistence of N‐coordinated Ru_1_ single atoms.

X‐ray absorption spectroscopy (XAS) was employed to thoroughly investigate the local coordination and electronic properties of the active centers. At the Fe K‐edge, the x‐ray absorption near‐edge structure (XANES) profile of Ru_NC_@Fe_1_NC strongly resembled that of a reference containing Fe single atoms, confirming the atomically dispersed state of Fe (Figure 2c) [21]. In the corresponding extended EXAFS spectrum, a primary peak emerged at approximately 1.6 Å, which was ascribed to Fe–N coordination [22]. This confirmed that the Fe atoms were exclusively coordinated to nitrogen functionalities in the carbon lattice (Figure 2d) [21]. Quantitative EXAFS fitting further resolved the first coordination shell as Fe–N with a coordination number of 3.7, approaching the fourfold coordination of the Fe–N_4_ moiety (Table S3). Wavelet transform (WT) EXAFS analysis yielded further support for this specific structural configuration (Figures S10 and S11).

**FIGURE 2:**
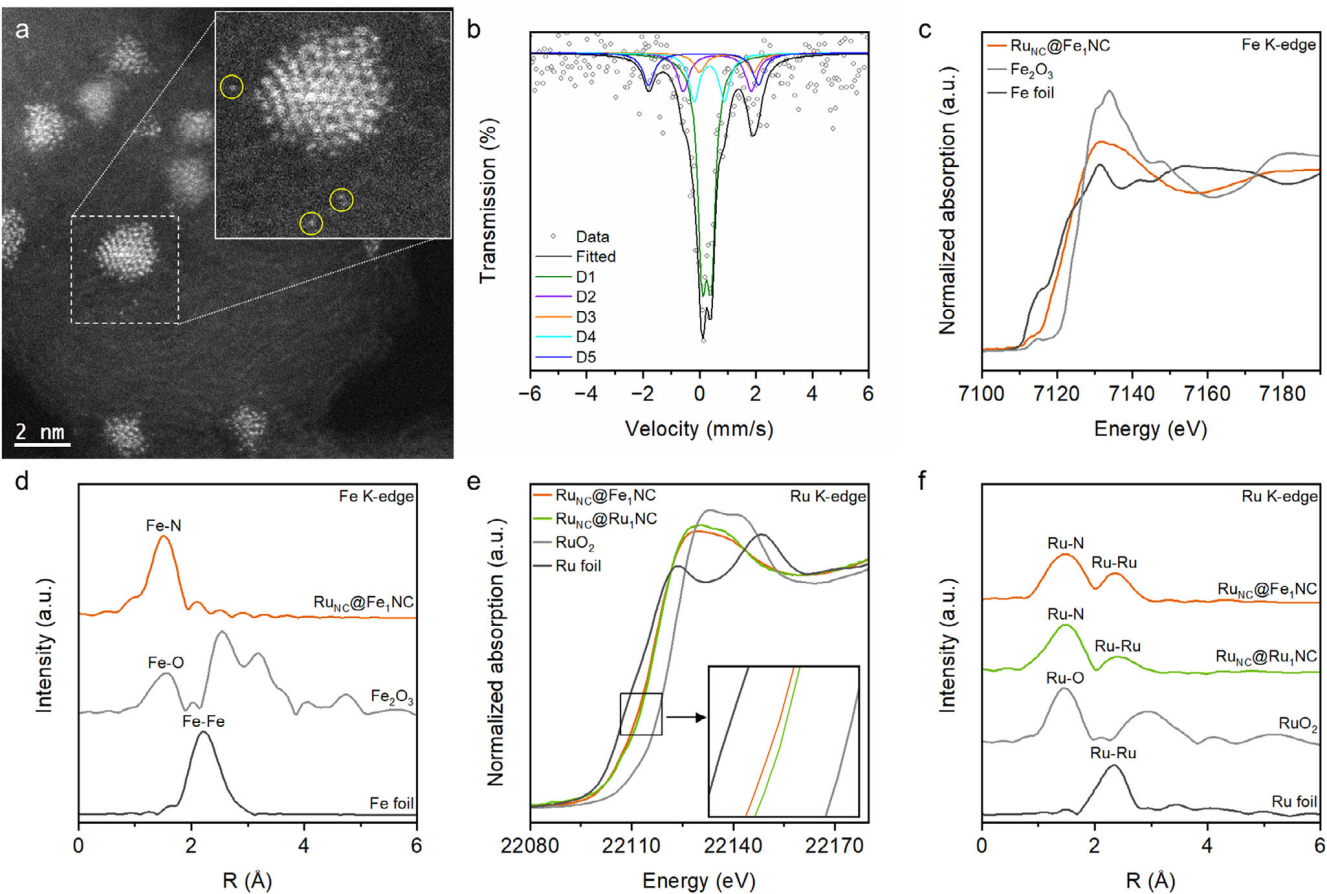
Atomic and electronic structure of the dual‐site Ru_NC_@Fe_1_NC catalyst. (a) Aberration‐corrected HAADF‐STEM image highlighting the atomically dispersed Fe single atoms (indicated by yellow circles). (b) ^57^Fe Mössbauer spectrum of Ru_NC_@Fe_1_NC. (c) Fe K‐edge XANES and (d) Fourier‐transformed EXAFS spectra of Ru_NC_@Fe_1_NC and reference samples. (e) Ru K‐edge XANES and (f) EXAFS spectra of Ru_NC_@Fe_1_NC, Ru_NC_@Ru_1_NC, and reference samples (inset in e shows enlarged pre‐edge region).

For the Ru K‐edge, both Ru_NC_@Fe_1_NC and Ru_NC_@Ru_1_NC showed XANES absorption thresholds positioned between the Ru foil and RuO_2_ references, pointing to the simultaneous presence of single atoms and metallic nanoclusters (Figure 2e) [23]. Importantly, relative to Ru_NC_@Ru_1_NC, the pre‐edge absorption of Ru_NC_@Fe_1_NC was shifted toward that of the Ru foil, implying a more electron‐enriched, reduced metallic nature for the Ru active sites. Such increased electron density is highly beneficial for the kinetics of the alkaline hydrogen evolution reaction (HER), optimizing both the adsorption of hydrogen and the ensuing evolution steps [24]. Additionally, the Ru K‐edge EXAFS data revealed a clear peak at ∼1.5 Å related to Ru─N bonds (single atoms), coupled with a strong peak near 2.4 Å reflecting Ru–Ru interactions within the nanoclusters (Figure 2f). These distinct dual‐site characteristics were also consistently observed in the WT‐EXAFS mapping (Figures S12 and S13).

### 2.2 Electrocatalytic Performances

The HER activities of Ru_NC_@Fe_1_NC and Ru_NC_@Ru_1_NC were systematically evaluated and benchmarked against commercial Ru/C and Pt/C catalysts in a 1.0 m aqueous KOH electrolyte under argon saturation. Linear sweep voltammetry (LSV) measurements were conducted to assess the intrinsic catalytic behavior (Figure 3a). The Ru_NC_@Fe_1_NC architecture substantially outperformed both the Fe‐free Ru_NC_@Ru_1_NC reference and the commercial benchmarks. This enhanced activity corresponded to significantly lower overpotentials required to drive practical current densities of 10, 20, and 30 mA cm^−2^ (Figure 3b). Notably, three specimens randomly sampled from different regions of the single ∼10 g batch delivered essentially identical polarization behaviors, further verifying the batch‐scale uniformity of the catalyst (Figure S14). In addition, the Fe_1_NC support alone exhibited negligible HER activity comparable to that of the bare carbon support, confirming that the oxophilic Fe_1_ sites cannot independently sustain hydrogen evolution in the absence of the adjacent Ru_NC_ (Figure S15).

**FIGURE 3:**
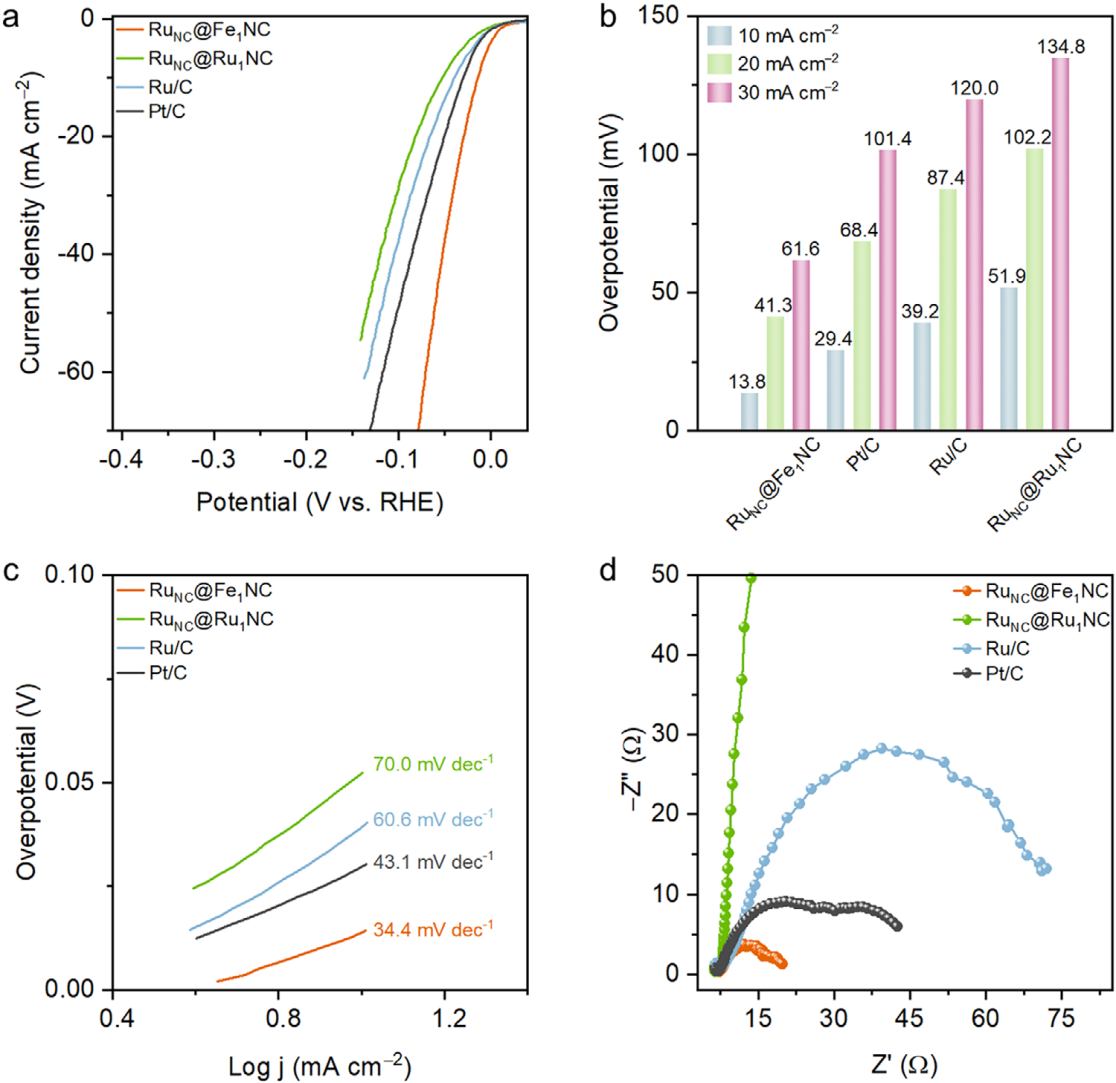
Electrocatalytic hydrogen evolution performance in 1.0 m aqueous KOH. (a) LSV polarization curves of Ru_NC_@Fe_1_NC, Ru_NC_@Ru_1_NC, commercial Ru/C, and Pt/C. (b) Overpotentials delivered at specific current densities. (c) Tafel plots. (d) Nyquist plots obtained from EIS measurements.

Kinetic evaluations derived from Tafel plots provided further evidence for this catalytic efficiency. Ru_NC_@Fe_1_NC yielded a Tafel slope of 34.4 mV dec^−1^, indicating highly accelerated reaction kinetics. This slope was substantially lower than those measured for Ru_NC_@Ru_1_NC (70.0 mV dec^−1^), Ru/C (60.6 mV dec^−1^), and Pt/C (43.1 mV dec^−1^) (Figure 3c). Notably, this exceptional kinetic performance was achieved despite an ultralow Ru loading of approximately 2 wt%, demonstrating a much higher mass‐specific utilization compared to the 20 wt% platinum content in commercial Pt/C. Electrochemical impedance spectroscopy (EIS) measurements were performed to gain insight into the interfacial charge‐transfer properties. At an overpotential of 10 mV, Ru_NC_@Fe_1_NC exhibited a significantly reduced charge‐transfer resistance compared with Pt/C, suggesting enhanced electron transport during HER (Figure 3d). These results highlighted the promotional role of oxophilic Fe single‐atom sites in facilitating alkaline HER kinetics. Furthermore, the electrochemically active surface area (ECSA) was evaluated through cyclic voltammetry (CV) measurements at various scan rates. Ru_NC_@Fe_1_NC showed the largest double‐layer capacitance (*C*_dl_), indicating a higher density of electrochemically accessible active sites compared to the other catalysts (Figures S16 and S17).

To further evaluate the intrinsic activity of the catalysts beyond the surface‐area effect, the iR‐corrected polarization currents were normalized by the ECSA derived from the double‐layer capacitance (Figure S18). Notably, even after the ECSA normalization, Ru_NC_@Fe_1_NC exhibited the highest specific activity among all the catalysts examined, confirming that its superior HER performance originates from the enhanced intrinsic activity of the Fe_1–_Ru_NC_ dual‐site architecture rather than from an enlarged electrochemically active surface area. The advantage of the ultralow Ru loading was further quantified by normalizing the polarization currents to the noble‐metal mass under identical low‐loading conditions (Figure S19). At an overpotential of 100 mV, Ru_NC_@Fe_1_NC delivered a mass activity of 13.7 A mg^−^^1^_metal_, approximately 12.6‐, 6.5‐, and 6.2‐fold higher than those of Ru_NC_@Ru_1_NC (1.09 A mg^−^^1^), commercial Pt/C (2.10 A mg^−^^1^), and Ru/C (2.21 A mg^−^^1^), respectively, highlighting the exceptional noble‐metal utilization efficiency of the dual‐site architecture.

To further examine the contributions of the metal sites, poisoning tests were conducted in a 1.0 m aqueous KOH electrolyte containing 10 mM ethylenediaminetetraacetic acid disodium salt (EDTA) or potassium thiocyanate (KSCN) [12, 25]. EDTA, a chelating agent for coordinatively accessible metal cations, exerted a negligible effect on the performance of Ru/C and Pt/C, whereas a pronounced activity decay was observed for Ru_NC_@Ru_1_NC and Ru_NC_@Fe_1_NC, both of which contain single‐atom metal species (Figure S20). This contrast indicates that EDTA acts preferentially on atomically dispersed sites and that these sites contribute substantially to catalytic activity. By comparison, the introduction of KSCN, a probe known to bind broadly to metal centers, resulted in severe performance degradation for all the catalysts, confirming that the observed activity originates from the metal sites rather than from the carbon support (Figure S21) [26]. These complementary results support that both the atomically dispersed species and the Ru_NC_ are critically involved in the overall HER activity of Ru_NC_@Fe_1_NC.

The durability of the Ru_NC_@Fe_1_NC catalyst was subsequently examined to evaluate its operational stability. Chronopotentiometry (CP) measurements conducted at a constant current density of −10 mA cm^−2^ for 24 h revealed excellent stability, with 98.1% of the initial activity retained. Furthermore, LSV curves recorded after 1000 continuous CV cycles showed negligible degradation compared to the initial polarization behavior, thereby confirming the robust electrochemical stability of Ru_NC_@Fe_1_NC under alkaline HER conditions (Figure S22).

To evaluate whether the intrinsic HER activity directly translates to practical operating environments, we assessed the catalysts within a full‐cell anion exchange membrane water electrolyzer (AEMWE) configuration. Membrane‐electrode assemblies (MEAs) were constructed by uniformly depositing the respective catalysts (Ru_NC_@Fe_1_NC or commercial Pt/C) onto nickel foam substrates via electrospray coating, followed by direct integration with an anion exchange membrane.

Cell polarization measurements revealed a vast performance gap between the dual‐site catalyst and the commercial benchmark. Specifically, the Ru_NC_@Fe_1_NC‐driven AEMWE cell achieved industrial‐scale current densities of 0.5 and 1.0 A cm^−2^ at cell voltages of merely 1.57 and 1.66 V, respectively. In sharp contrast, the Pt/C‐based device required significantly higher operating voltages of 1.77 and 1.93 V to reach identical current targets (Figure 4a). This substantial voltage reduction confirmed the superior catalytic proficiency of Ru_NC_@Fe_1_NC as a cathode in practical electrolyzer systems.

**FIGURE 4:**
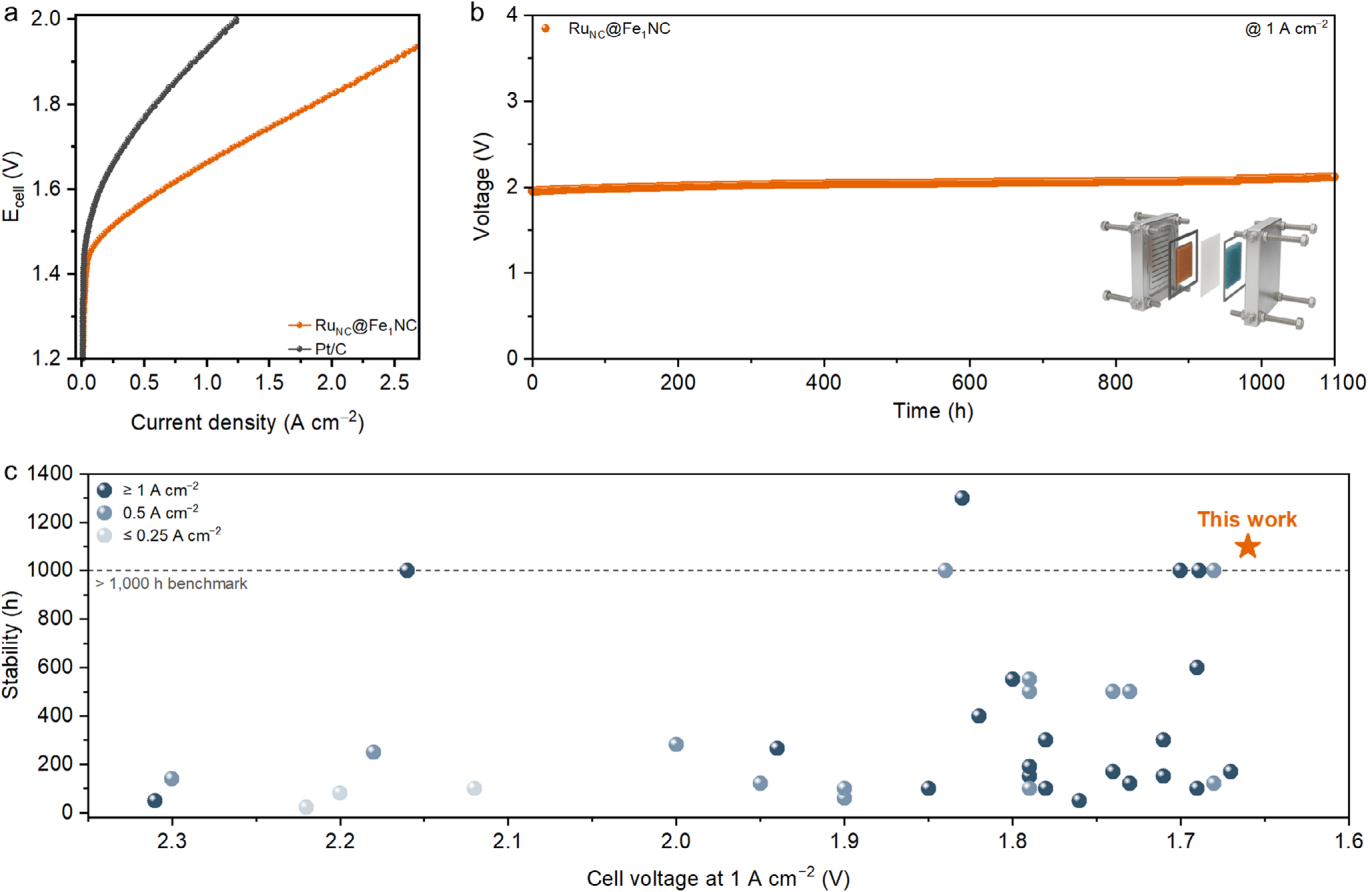
Device‐level performance in an anion‐exchange membrane water electrolyzer (AEMWE). (a) Polarization curves of the AEMWE cells employing Ru_NC_@Fe_1_NC and commercial Pt/C as cathode catalysts. (b) Long‐term chronopotentiometry (CP) stability test of Ru_NC_@Fe_1_NC at an industrial current density of 1.0 A cm^−^^2^ and 80°C (inset shows the schematic of the integrated AEMWE full‐cell configuration). (c) Comparison of the cell voltage at 1.0 A cm^−^^2^ and the long‐term stability of Ru_NC_@Fe_1_NC with recently reported AEMWE cells.

Beyond initial activity, the operational longevity of the catalyst was rigorously tested via chronopotentiometry. When subjected to a severe current density of 1.0 A cm^−^^2^, the Ru_NC_@Fe_1_NC‐integrated AEMWE cell operated continuously for over 1100 h with negligible voltage fluctuation (Figure 4b). To verify the structural origin of this long‐term durability, post‐mortem analyses were performed on the cathode recovered after the 1100 h operation. At the electrode level, the catalyst coating layer remained firmly attached to the nickel foam substrate without visible delamination or detachment after the prolonged operation (Figure S23). Atomic‐resolution HAADF‐STEM images revealed that the Fe single atoms remain atomically dispersed without any sign of aggregation, while the Ru nanoclusters preserve their original sizes and dispersion (Figure S24). EDS elemental mapping further confirmed the uniform distribution of C, N, Ru, and Fe throughout the catalyst, verifying that the dual‐site architecture is robustly maintained under prolonged AEMWE operation (Figure S25). Together, the stable 1100 h operation and the intact post‐operation structure underscore the exceptional electrochemical resilience of the Ru_NC_@Fe_1_NC architecture.

The device‐level efficacy of Ru_NC_@Fe_1_NC was further benchmarked against recently published AEMWE systems in terms of both activity and durability. Operating at a high current demand of 1.0 A cm^−^^2^, the cell voltage required by the Ru_NC_@Fe_1_NC device positioned it among the lowest energy‐input records documented in the literature under similar testing parameters (Figure S26 and Table S4). More importantly, among the reported electrocatalysts, Ru_NC_@Fe_1_NC uniquely combines the lowest cell voltage at 1.0 A cm^−^^2^ with a stability exceeding 1000 h under the demanding current density of 1.0 A cm^−^^2^, positioning it in the most favorable region of the activity–durability landscape (Figure 4c). This rigorous system‐level comparison demonstrated the distinct capability of Ru_NC_@Fe_1_NC to drive highly energy‐efficient and durable alkaline water splitting. Ultimately, the successful convergence of top‐tier full‐cell efficiency, exceptional long‐term stability, and proven gram‐scale synthesizability established Ru_NC_@Fe_1_NC as a highly promising candidate for industrial alkaline water electrolysis.

### 2.3 Mechanistic Insights Into Alkaline HER

We investigated the underlying water activation mechanisms to explicitly decouple the distinct catalytic roles of the single‐atom sites (Fe_1_ versus Ru_1_) while holding the Ru_NC_ constant. Carbon monoxide (CO) stripping analysis was first employed to evaluate the surface hydroxyl (OH^−^) affinity, where a lower oxidation potential signifies stronger OH^−^ adsorption and accelerated water dissociation kinetics (Figure 5a) [27]. The dual‐site Ru_NC_@Fe_1_NC architecture registered a conspicuously lower CO oxidation potential compared to both the Fe‐free Ru_NC_@Ru_1_NC reference and commercial Ru/C (Figure S27). This distinct potential shift confirmed that the Fe_1_ centers possessed substantially higher oxophilicity than the Ru_1_ sites, thereby facilitating the critical OH^−^ adsorption step during the alkaline reaction.

**FIGURE 5:**
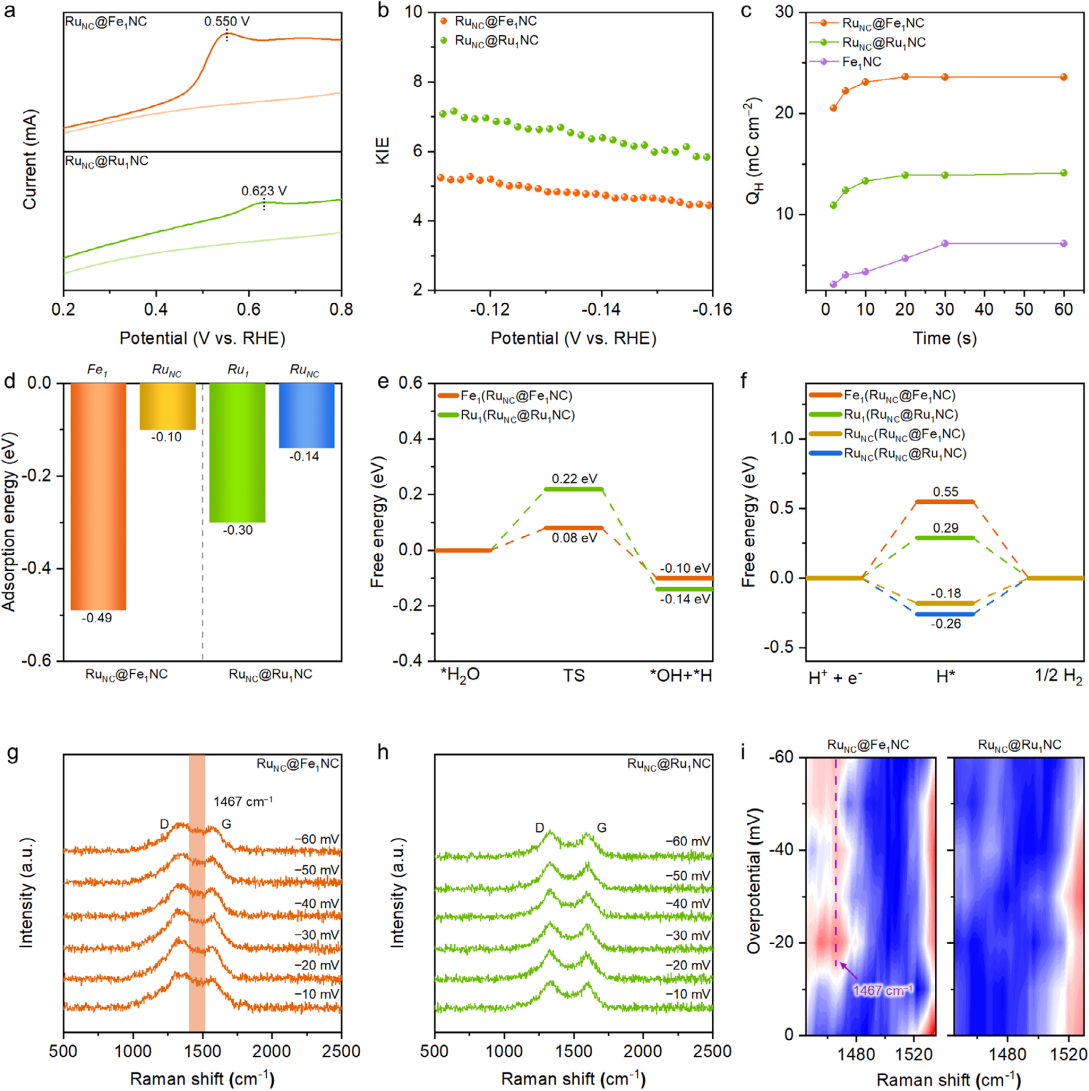
Mechanistic insights into the alkaline hydrogen evolution reaction (HER) on the Ru_NC_@Fe_1_NC catalyst. (a) Carbon monoxide stripping voltammograms of Ru_NC_@Fe_1_NC and Ru_NC_@Ru_1_NC. (b) Kinetic isotope effect (KIE) profiles in H_2_O‐ and D_2_O‐based alkaline electrolytes. (c) Accumulated hydrogen charge (*Q*_H_) as a function of accumulation time for Ru_NC_@Fe_1_NC, Ru_NC_@Ru_1_NC, and Fe_1_NC, obtained from time‐resolved hydrogen accumulation–stripping measurements. (d) Calculated H_2_O adsorption energies on different active sites. (e) Free energy diagrams for the water dissociation process. (f) Calculated free energy diagrams of hydrogen adsorption on different active sites. Note that Fe_1_, Ru_1_, and Ru_NC_ denote the Fe single‐atom site, Ru single‐atom site, and Ru nanocluster site, respectively. In situ Raman spectra obtained for (g) Ru_NC_@Fe_1_NC and (h) Ru_NC_@Ru_1_NC, and (i) the corresponding potential‐resolved Raman contour plot.

Isotopic experiments utilizing H_2_O‐ and D_2_O‐based electrolytes served to further quantify the water dissociation kinetics by extracting the kinetic isotope effect (KIE) (Figure 5b) [14]. All evaluated materials produced KIE values above unity, verifying that O─H bond cleavage governed the overall reaction rate. Crucially, the Ru_NC_@Fe_1_NC system yielded a significantly suppressed KIE value relative to its Ru_NC_@Ru_1_NC counterpart. This suppressed isotopic dependence demonstrated a lowered kinetic barrier for water dissociation. Collectively, these results established that integrating highly oxophilic Fe_1_ sites alongside Ru_NC_ fundamentally accelerated the initial O─H bond cleavage step relative to systems relying solely on Ru_1_ centers.

To experimentally trace the fate of the hydrogen intermediates generated from water dissociation, we further performed time‐resolved surface hydrogen accumulation–stripping analysis (Figure 5c) [28]. In these measurements, the electrode was held at a mild cathodic potential to allow H* to accumulate on the catalyst surface for controlled durations, and the accumulated hydrogen charge (*Q*_H_) was immediately quantified by an anodic stripping sweep, in which the integrated charge of the *t* = 0 baseline was subtracted to cancel the capacitive contributions (Figure S28). Ru_NC_@Fe_1_NC exhibited both the fastest accumulation kinetics, reaching approximately 94% of its saturation value within 5 s, and the highest saturated charge (*Q*_sat_ = 23.6 vs. 14.1 mC cm^−^^2^ for Ru_NC_@Ru_1_NC), whereas Fe_1_NC remained at the background level (≤7.1 mC cm^−^^2^), confirming that the Fe_1_ sites alone cannot store the generated hydrogen. Moreover, the desorption charge obtained from the stripping curves remained essentially constant (17.3 ± 0.8 mC cm^−^^2^) over the 20‐fold scan‐rate range of 10–200 mV s^−^^1^, in clear contrast to the approximately 4.5‐fold decrease expected for a diffusion‐controlled process, verifying that the measured signals originate from surface‐adsorbed H* rather than from diffusing species such as dissolved H_2_ (Figures S29 and S30) [29]. Considering the comparable Ru loadings and cluster sizes of the two dual‐site catalysts, the markedly faster and larger accumulation of H* on Ru_NC_@Fe_1_NC can only be attributed to water dissociation occurring at the Fe_1_ sites, followed by the transfer of the generated hydrogen to the adjacent Ru_NC_, providing direct electrochemical evidence for the proposed Fe_1_‐to‐Ru_NC_ hydrogen supply.

Density functional theory (DFT) calculations were performed to theoretically elucidate the superior alkaline HER activity of the catalysts with coexisting Fe_1_ and Ru_NC_. To this end, models representing the dual‐site architecture with coexisting Fe_1_ and Ru_NC_ (Ru_NC_@Fe_1_NC) as well as a control structure containing Ru_1_ and Ru_NC_ (Ru_NC_@Ru_1_NC) were constructed (Figure S31). To assess the water‐trapping capability in the alkaline HER environment, the water adsorption energies for each structure were calculated (Figure 5d). For each coexisting catalyst, two distinct active sites were separately considered. The first is the single‐atom site (Fe_1_ (Ru_NC_@Fe_1_NC) or Ru_1_ (Ru_NC_@Ru_1_NC)) and the second is the Ru nanocluster site (Ru_NC_ (Ru_NC_@Fe_1_NC) or Ru_NC_ (Ru_NC_@Ru_1_NC)). The calculations revealed that for both structures, the single‐atom sites exhibited stronger water adsorption energies compared to the adjacent Ru_NC_ sites. Notably, the Fe_1_ site displayed significantly stronger adsorption energy (−0.49 eV) than the Ru_1_ site (−0.30 eV), indicating that the Fe_1_ center dictates the primary interaction with H_2_O within the Ru_NC_@Fe_1_NC catalyst. Subsequently, the free energy diagrams for the water dissociation process on the Fe_1_ and Ru_1_ sites were calculated (Figure 5e). The results demonstrated that the dissociated states are thermodynamically stable in both systems. Furthermore, the activation barrier on Fe_1_ (Ru_NC_@Fe_1_NC) is approximately 0.08 eV, confirming that it is kinetically far more favorable than the 0.22 eV barrier on Ru_1_ (Ru_NC_@Ru_1_NC). These findings indicate that Fe_1_ is expected to exhibit superior catalytic performance compared to Ru_1_ during the Volmer step.

In the alkaline HER process, the adsorption behavior of the *OH intermediate generated after water dissociation is a critical factor determining the overall HER performance. The OH adsorption energies were calculated on the single‐atom sites, which dominate the water adsorption and dissociation performance (Figure S32). The calculations revealed that the Fe_1_ (Ru_NC_@Fe_1_NC) site exhibits a stronger *OH adsorption energy compared to the Ru_1_ (Ru_NC_@Ru_1_NC) site. This indicates that the Fe_1_ site exhibits a tighter binding affinity than the Ru_1_ site, allowing it to effectively control the local environment. As a result, the stable anchoring of the *OH species at the Fe_1_ site is expected to induce the dissociated hydrogen atom to favorably migrate to the adjacent Ru_NC_ site.

Furthermore, to investigate the stronger H_2_O and OH adsorption tendencies of the Fe_1_ site compared to the Ru_1_ site from an electronic perspective, a Mulliken charge analysis was performed on each adsorption structure (Figure S33). A comparison of the charge distributions in the optimized structures revealed that the Fe_1_ site consistently exhibited a higher positive charge, while the adsorbed oxygen atom exhibited a more negative charge than that observed on the Ru_1_ site. The charge separation between the metal and oxygen species was significantly greater in the Fe–O interaction than in the Ru–O interaction, indicating a more strongly polarized metal–oxygen bond and enhanced electron transfer.

To evaluate the feasibility of hydrogen desorption during the alkaline HER process, the hydrogen adsorption free energies (Δ*G*_H*_) were calculated for the single‐atom and nanocluster sites of each catalyst structure (Figure 5f). The single‐atom sites of both catalyst structures exhibited positive Δ*G*_H*_ values, indicating that hydrogen adsorption is thermodynamically unfavorable at these sites. Accordingly, the hydrogen atom generated from water dissociation is expected to migrate to the adjacent Ru_NC_ site rather than remain on the single‐atom site, in excellent agreement with the accumulation–stripping observations (Figure 5c). In contrast, the Ru_NC_ sites of both catalyst structures exhibited negative Δ*G*_H*_ values and thus are expected to serve as thermodynamically favorable active sites for hydrogen adsorption and desorption. Notably, the Δ*G*_H*_ of the Ru_NC_ site changed upon substituting the single atom from Ru_1_ to Fe_1_. The Ru_NC_ (Ru_NC_@Ru_1_NC) site exhibited a rather strong hydrogen binding strength of −0.26 eV, whereas after the Fe_1_ substitution, the adjacent Ru_NC_ (Ru_NC_@Fe_1_NC) site exhibited a more optimized value of −0.18 eV, which is closer to the thermoneutral point. These results suggest the presence of a synergistic effect, in which the introduction of Fe_1_ optimizes the balance between hydrogen adsorption and desorption at the Ru_NC_ site, thereby facilitating the hydrogen desorption process.

Finally, to directly monitor the water‐capturing behavior of the Fe_1_ sites, in situ Raman spectroscopy was employed under operando alkaline HER conditions (Figure 5g,h). Ru_NC_@Fe_1_NC exhibited a distinct Raman band centered at approximately 1467 cm^−^^1^, which precisely matches the theoretically predicted Raman shift for the H_2_O‐adsorbed configuration on the Fe_1_ site, as validated by our density functional theory (DFT) calculations (Table S5). In contrast, Ru_NC_@Ru_1_NC displays only minimal spectral changes across the applied potential range, suggesting a much weaker interaction with H_2_O molecules. This difference is further highlighted in the potential‐resolved Raman contour plot (Figure 5i), which clearly visualizes the progressive emergence of the 1467 cm^−^^1^ feature on Ru_NC_@Fe_1_NC with increasing cathodic polarization, corroborating the highly oxophilic nature of the Fe_1_ sites for capturing water molecules during the Volmer step.

In summary, the Fe_1_ (Ru_NC_@Fe_1_NC) site dominates the water dissociation process owing to its strong oxophilicity, and the generated hydrogen intermediates are rapidly supplied to the adjacent Ru_NC_ (Ru_NC_@Fe_1_NC) site, which effectively facilitates the subsequent hydrogen desorption and evolution, as evidenced both electrochemically and theoretically. Therefore, this synergistic effect between the Fe single atoms and the Ru nanoclusters is expected to contribute to the excellent HER performance (Figure S34).

## 3 Conclusion

In summary, we demonstrate a scalable, solvent‐free mechanochemical strategy utilizing high‐energy impact to construct a heterogeneous dual‐site electrocatalyst (Ru_NC_@Fe_1_NC) for industrial alkaline hydrogen production. This approach anchors isolated oxophilic Fe_1_ centers within a defective carbon host, followed by the targeted integration of Ru nanoclusters. Ru_NC_@Fe_1_NC delivers accelerated alkaline HER kinetics, requiring an overpotential of merely 13.8 mV to reach 10 mA cm^−2^ with an ultralow Ru loading (∼2 wt%), substantially outperforming commercial Pt/C and Ru/C benchmarks. Practically, the Ru_NC_@Fe_1_NC‐integrated AEMWE cell achieves an industrial current density of 1.0 A cm^−2^ at 1.66 V, sustaining continuous operation for over 1100 h with negligible decay. Crucially, comprehensive analyses—including isotopic substitution, CO stripping, in situ Raman spectroscopy, time‐resolved hydrogen accumulation–stripping measurements, and DFT calculations—established a definitive mechanistic framework. They decoupled the distinct roles of the active sites: highly oxophilic Fe_1_ centers selectively bind water to lower the kinetic barrier for initial O─H bond cleavage (Volmer step), while adjacent Ru nanoclusters provide optimal energetics for rapid H–H recombination—a division of labor directly evidenced by the electrochemically quantified Fe_1_‐to‐Ru_NC_ hydrogen transfer. This spatial decoupling effectively mitigates the intrinsic kinetic bottlenecks of alkaline environments. The successful convergence of 10‐gram scale manufacturability, mechanistic clarity, and exceptional full‐cell longevity positions Ru_NC_@Fe_1_NC as a compelling candidate for commercial anion exchange membrane water electrolyzers.

## 4 Experimental Section/Methods

### 4.1 Preparation of Fe_1_NC

The Fe_1_NC support was synthesized by a mechanochemical ball‐milling process using a planetary mill (Pulverisette 6, Fritsch). Graphite powder (20.0 g, 100 mesh, 99.9995%, Alfa Aesar) and hardened steel balls (500 g, diameter 5 mm, 99 wt% Fe) were loaded into a hardened steel vessel (250 mL). Prior to milling, the vessel was evacuated and purged with high‐purity N_2_ gas (99.999%, N50, KOSEM) at least three times, followed by pressurization to 10 bar. Ball milling was conducted at 500 rpm for 48 h. To prevent excessive temperature rise, the milling process was paused for 10 min after every 60 min of operation. After milling, the residual N_2_ gas was slowly released under ambient conditions. The obtained powder was subsequently annealed at 850°C under an Ar atmosphere.

### 4.2 Preparation of Ru_NC_@Fe_1_NC

Ruthenium was deposited onto the Fe_1_NC support via a wet impregnation method. Ruthenium(III) chloride hydrate (RuCl_3_·xH_2_O, 2.57 g) was dissolved in NMP (1 L). The solution was ultrasonicated for 15 min in an ice bath to ensure complete dissolution. The Fe_1_NC support (9.5 g) was then added to the Ru precursor solution and stirred for 24 h at room temperature. After impregnation, the solid product was collected by filtration and washed repeatedly with ethanol. The resulting powder was dried in a vacuum oven at 60°C.

### 4.3 Preparation of Ru_NC_@Ru_1_NC

The Ru_NC_@Ru_1_NC catalyst was prepared following the same procedure as that used for Ru_NC_@Fe_1_NC, except for the synthesis conditions of the nitrogenated carbon (NC) support. Specifically, the ball‐milling process for the NC support was carried out at a reduced rotational speed of 300 rpm, while all other experimental parameters and post‐treatment steps remained identical. The subsequent Ru impregnation, washing, and drying procedures were performed following the same protocol described for Ru_NC_@Fe_1_NC.

### 4.4 Characterization

The metal contents of the catalysts were quantified by inductively coupled plasma–optical emission spectroscopy (ICP–OES, Optima 5300 DV, PerkinElmer). High‐angle annular dark‐field scanning transmission electron microscopy (HAADF–STEM) images were acquired using a JEM‐ARM300F microscope equipped with a field‐emission gun and operated at an accelerating voltage of 300 kV. X‐ray absorption spectroscopy (XAS) measurements were performed at the 6D UNIST–PAL beamline of the Pohang Light Source (PSL‐II, Korea). The obtained spectra were processed and analyzed using the ATHENA software package. X‐ray photoelectron spectroscopy (XPS) measurements were conducted on a K‐Alpha spectrometer (Thermo Fisher Scientific, UK). Elemental analyses were carried out using a Flash 2000 elemental analyzer. X‐ray diffraction (XRD) patterns were collected on a high‐power diffractometer (D/MAX 2500 V/PC, Rigaku, Japan) with Cu Kα radiation (*λ* = 1.5418 Å) operated at 35 kV and 20 mA. ^57^Fe Mössbauer spectroscopy was performed at room temperature using a ^57^Co (Rh) source (Palacký University, 01216CN). Spectra were recorded in transmission geometry with a triangular velocity waveform.

### 4.5 Electrochemical Measurements

Electrochemical measurements were performed using an electrochemical workstation (Ivium, Netherlands) in a standard three‐electrode configuration, with a graphite rod as the counter electrode and an Ag/AgCl electrode (saturated KCl) as the reference electrode. All measured potentials were converted to the reversible hydrogen electrode (RHE) scale. For catalyst ink preparation, 5 mg of catalyst was dispersed in a mixed solvent containing isopropyl alcohol (980 µL) and Nafion solution (20 µL, DuPont), followed by ultrasonication in an ice bath for 30 min and subsequent overnight stirring at room temperature to obtain a homogeneous suspension. A defined volume (30 µL) of the catalyst ink was drop‐cast onto a rotating ring–disk electrode (RRDE, disk diameter: 4 mm) to form a uniform catalyst layer. Linear sweep voltammetry (LSV) was conducted in 1.0 m aqueous KOH at room temperature using a scan rate of 5 mV s^−1^ and a rotation speed of 1600 rpm. All polarization curves were corrected for ohmic losses (iR correction). Electrochemical impedance spectroscopy (EIS) measurements were carried out in the same electrolyte by applying a sinusoidal AC voltage with an amplitude of 10 mV over a frequency range from 0.1 to 10^5^ Hz.

Tafel plots were derived from steady‐state polarization curves obtained from LSV measurements and analyzed using the Tafel equation, *η* = *b* log *j* + *a*, where *η* is the overpotential, *b* is the Tafel slope, and *a* is a constant; the Tafel slope was extracted from the linear region of the *η* versus log *j* plot. The electrochemical double‐layer capacitance (*C*_dl_) was estimated from cyclic voltammetry (CV) measurements performed within a non‐faradaic potential region at scan rates of 20, 40, 60, 80, and 100 mV s^−^^1^, with *C*_dl_ values obtained from the slope of the linear fit between capacitive current and scan rate. To probe the nature of the catalytic active sites, poisoning experiments were conducted using an electrolyte containing 10 mM EDTA and 10 mM KSCN in 1.0 m aqueous KOH under otherwise identical conditions to those used for LSV measurements.

Catalyst stability was evaluated by chronopotentiometry (CP) and cyclic voltammetry (CV). CP measurements were performed at a constant current density of −10 mA cm^−2^ while monitoring the corresponding potential variation over time. Accelerated durability tests were further carried out by continuous CV cycling between 0.05 and 1.0 V versus RHE at a scan rate of 100 mV s^−1^ for 1000 cycles, after which LSV curves were recorded to assess performance degradation. CO stripping experiments were conducted in 1.0 m aqueous KOH to investigate CO adsorption behavior, during which the working electrode was held at 0.05 V versus RHE while a CO/Ar gas mixture was bubbled through the electrolyte for 10 min, followed by Ar purging for an additional 10 min without applied potential; CO stripping voltammograms were subsequently recorded by LSV at a scan rate of 5 mV s^−1^. The specific activity was evaluated by normalizing the iR‐corrected LSV currents by the electrochemically active surface area (ECSA). The ECSA was calculated according to ECSA = *C*_dl_/*C*_s_, where *C*_dl_ is the double‐layer capacitance obtained from the scan‐rate‐dependent CV measurements and *C*_s_ is the specific capacitance of a flat surface. The mass activity was evaluated by normalizing the iR‐corrected LSV currents to the noble‐metal mass loaded on the electrode. The metal mass was calculated from the catalyst loading and the Ru contents determined by ICP‐OES for Ru_NC_@Fe_1_NC and Ru_NC_@Ru_1_NC, and commercial catalysts (20 wt% for Pt/C and 5 wt% for Ru/C).

Time‐resolved surface hydrogen accumulation–stripping analysis was performed in a standard three‐electrode configuration using an RDE, with a graphite rod as the counter electrode and an Ag/AgCl (saturated KCl) electrode as the reference electrode, in Ar‐saturated 1.0 m aqueous KOH. Prior to the measurements, the electrode surface was conditioned by cyclic voltammetry between 0.05 and 0.6 V versus RHE until stable voltammograms were obtained. For hydrogen accumulation, the electrode was held at a mild overpotential of 20 mV, at which the hydrogen evolution reaction is just initiated, for controlled durations of 2–60 s. Immediately after each holding step, an anodic linear sweep was recorded from 0.05 to 0.6 V versus RHE at 50 mV s^−^^1^ to oxidatively strip the accumulated hydrogen. All the stripping voltammograms are presented as measured, without any correction. For the quantification, a baseline sweep recorded without a holding step (*t* = 0) was additionally collected; because the double‐layer charging current is independent of the holding time, the accumulated hydrogen charge (*Q*_H_) was obtained by integrating each stripping curve and subtracting the integrated charge of the *t* = 0 baseline, which cancels the capacitive and background contributions. Electrode rotation (1600 rpm) was applied during the holding step to remove the evolved H_2_ from the electrode surface, and the rotation was stopped immediately before each anodic sweep. Continuous Ar purging was maintained throughout the measurements to minimize interference from the oxidation of dissolved H_2_, and identical cleaning cycles were applied between all measurements to ensure a reproducible initial surface state. For the scan‐rate‐dependent measurements, the accumulation time was fixed at 60 s, and the stripping sweeps were recorded at scan rates of 10, 20, 30, 40, 50, 100, and 200 mV s^−^^1^. In this case, the desorption charge (Q) was determined by integrating each stripping curve after subtracting the capacitive background current measured at 0.6 V versus RHE and dividing the integrated area by the corresponding scan rate.

### 4.6 AEM Water Electrolysis Performance

Catalyst inks were prepared by dispersing 30 mg of catalyst in a mixed solvent of deionized water and isopropyl alcohol (IPA) with a volume ratio of 1:3, yielding a total volume of 15 mL. Nafion solution was added to achieve an ionomer‐to‐carbon (I/C) ratio of 0.4. The resulting suspension was ultrasonicated in an ice bath for 1 h to obtain a homogeneous ink. The catalyst ink was subsequently deposited onto nickel foam (NF) substrates via an electrospray technique. For this process, the ink was loaded into a plastic syringe fitted with a 30‐gauge stainless steel needle and connected to a high‐voltage power supply (ESN‐HV30). A voltage of 5.0–6.0 kV was applied between the needle and the NF substrate, which were separated by 8.5 cm, while the ink was delivered at a controlled flow rate of 75 µL min^−1^ using a syringe pump (KD Scientific, Model 220).

The catalyst loadings were controlled to approximately 0.1 mg_Ru_ cm^−2^ for Ru_NC_@Fe_1_NC and 0.2 mg_Pt_ cm^−2^ for Pt/C on the cathode, while an IrO_2_ loading of approximately 1 mg cm^−2^ was employed for the anode. The anion exchange membrane water electrolyzer (AEMWE) was assembled by sandwiching the cathode, anion exchange membrane, and anode between two titanium bipolar plates and tightening to a torque of 10 N·m. A commercial anion exchange membrane (Sustainion X37‐50, dioxide materials) was used for polarization (*I*–*V*) measurements, whereas a different membrane (FAA‐3‐PK‐75, Fumasep) was employed for stability tests. Prior to use, the membranes were immersed in 1.0 m aqueous KOH solution for at least 24 h to ensure full ion exchange. The AEMWE cell was operated at 80°C with 1.0 m aqueous KOH circulated through the anodic compartment at a flow rate of 20 mL min^−1^ using a peristaltic pump (YZ1515, Longer Pump). All electrochemical measurements of the AEMWE were conducted without iR correction.

### 4.7 Kinetic Isotope Effect Measurement

Kinetic isotope effect (KIE) measurements were performed in 1.0 m aqueous KOH prepared with H_2_O and in 1.0 m KOD prepared with D_2_O, respectively. The current densities obtained at identical overpotentials in the H‐ and D‐containing electrolytes were denoted as *j*_H_ and *j*_D_. The KIE value was determined from the ratio of the two current densities according to:

$$\mathrm{KIE}=\frac{j_{H}}{j_{D}}$$

### 4.8 In Situ Raman Spectroscopy

In situ Raman spectroscopy measurements were performed using a confocal Raman microscope equipped with a 50x objective lens and a 633 nm laser, within a homemade electrochemical Raman cell filled with 1.0 m aqueous KOH electrolyte. Ru_NC_@Fe_1_NC and Ru_NC_@Ru_1_NC catalysts were drop‐cast onto a gas diffusion electrode (GDE, Sigracet 39BB) and employed as the working electrode. A dimensionally stable anode (DSA) and a Hg/HgO electrode (1.0 m aqueous KOH) were used as the counter and reference electrodes, respectively. For visualization of potential‐dependent spectral evolution, Raman contour plots were generated using an identical spectral window and baseline‐adjusted to highlight relative changes in the Raman features.

### 4.9 Calculation Details

To investigate the bifunctional alkaline hydrogen evolution reaction (HER) on the coexisting Fe single atom and Ru nanocluster catalyst, density functional theory (DFT) calculations were performed using the Cambridge Serial Total Energy Package (CASTEP) program [30]. The generalized gradient approximation (GGA) with the Perdew–Burke–Ernzerhof (PBE) exchange–correlation functional was used [31]. To describe the dispersion interactions, the Grimme scheme was employed [32]. The electron–ion interaction was described by ultrasoft pseudopotentials with a plane‐wave basis set cutoff energy of 350 eV [33]. The Brillouin zone was sampled by a 3 × 3 × 1 k‐point set with the Monkhorst–Pack scheme [34]. To account for the strong on‐site Coulomb interaction of the Fe 3d electrons, a Hubbard U correction was applied with *U* = 5.3 eV. Geometry optimization was carried out using the two‐point steepest descent (TPSD) algorithm until the total energy, maximum force, and maximum displacement converged below 1.0 × 10^−5^ eV/atom, 0.03 eV/Å, and 1.0 × 10^−3^ Å, respectively [35].

The N‐doping formation energy of graphene (i.e., Δ*E*_Df_) was calculated according to the following equation:

$$\Delta E_{\mathrm{Df}}=E_{\mathrm{doped}}\;-\;E_{\mathrm{pristine}}+n_{C}E_{C}\;-\;n_{N}E_{N}\;-\;n_{M}E_{M}$$

where *E*_doped_ and *E*_pristine_ represent the total energies of the doped and pristine graphene, respectively, and *E*_C_, *E*_N_, and *E*_M_ represent the energies of the reference states of carbon, nitrogen, and the metal atom (i.e., Fe or Ru), respectively. *n*_C_, *n*_N_, and *n*_M_ denote the numbers of the corresponding atoms removed from or added to the pristine graphene upon doping. The binding energy between the Ru nanocluster and the N‐doped graphene (i.e., Δ*E*_binding_) was calculated according to the following equation:

$$\Delta E_{\mathrm{binding}}=E_{\mathrm{total}}\;-\;E_{\mathrm{Ru}}\;-\;E_{\mathrm{doped}}$$

where *E*_total_ represents the total energy of the Ru nanocluster supported on the N‐doped graphene, and *E*_Ru_ and *E*_doped_ represent the energies of the Ru nanocluster and the N‐doped graphene, respectively. The adsorption energies (i.e., Δ*E*_ads_) of *H_2_O and *OH were calculated according to the following equation:

$$\Delta E_{\mathrm{ads}}=E_{\mathrm{total}}\;-\;\left(E_{\mathrm{adsorbate}}+E_{\mathrm{surface}}\right)$$

where *E*_total_ represents the total energy of the surface with the adsorbate, and *E*_adsorbate_ and *E*_surface_ represent the energies of the adsorbate and the surface, respectively. The H‐adsorption free energy change (i.e., Δ*G*_H*_) was calculated according to the following equation:

$$\Delta G_{H^{*}}=\;\Delta E_{\mathrm{ads}}+\;\Delta \mathrm{ZPE}\;-\;T\Delta S$$

where ΔZPE, *T*, and Δ*S* represent the change in zero‐point energy, the absolute temperature, and the entropy change of hydrogen adsorption at room temperature, respectively. The catalyst structures were modeled on a nitrogenated carbon (NC) substrate (i.e., N‐doped graphene). A graphene surface slab was constructed as a 6 × 6 × 1 supercell, which was sufficiently large to accommodate a Ru_13_ nanocluster, and a vacuum region greater than 30 Å was introduced in the z‐direction. The main system consisted of an Fe single atom and a Ru nanocluster on the NC (i.e., Ru_NC_@Fe_1_NC), and the comparative system consisted of a Ru single atom and a Ru nanocluster on the NC (i.e., Ru_NC_@Ru_1_NC). Based on the experimental XAFS spectra analysis (Figure 2c–f and Table S3), the Fe‐N_4_ and Ru‐N_4_ functional groups were considered for the nitrogen (N) sites bonded with the Fe single atom and the Ru single atom, and the graphitic‐N functional group was considered for the N sites bonded with the Ru nanocluster. The most thermodynamically stable nitrogen doping site was determined through the doping formation energy (Figure S35), and the most stable catalyst structure was modeled by calculating the binding energy between the Ru nanocluster and the N‐doped graphene (Figure S36).

## Author Contributions

J.‐H.B. designed the overall experiments and carried out the catalyst synthesis, materials characterization, and electrochemical measurements, and wrote the manuscript. S.H.K. performed the density functional theory (DFT) calculations. S.G.C. conducted the in situ Raman spectroscopy measurements. S.J.L. assisted with the materials synthesis. J.B. assisted with the ICP‐OES measurements. D.H.K. assisted with the DFT calculations. H.L. assisted with the in situ Raman spectroscopy measurements. Y.K. supervised the in situ Raman spectroscopy studies. S.K.K. supervised the DFT calculations. J.‐B.B. conceived and supervised the project and revised the manuscript.

## Conflicts of Interest

The authors declare no conflicts of interest.

## Supporting information




**Supporting File**: adma74980‐sup‐0001‐SuppMat.docx.

## Data Availability

The datasets generated and analyzed during this study are available from the corresponding author upon reasonable request.
