# Supplementary material for: Atom–Cluster Synergy in Scalable Fe–Ru Dual‐Site Architectures Accelerates Alkaline Hydrogen Evolution
Source: Adv Mater. 2026 Sep 10;38(60):e74980. doi: 10.1002/adma.74980 (PMC13712209; doi:10.1002/adma.74980)
Supplement: Supplementary file 1 [file ADMA-38-e74980-s001.docx]

Supporting Information

Atom–Cluster Synergy in Scalable Fe–Ru Dual-Site Architectures Accelerates Alkaline Hydrogen Evolution

Jae-Hoon Baek,^#^ Seong Hyeon Kweon,^#^ Sun Gwan Cha,^#^ Se Jung Lee, Jinwoo Baek, Dong Hyeok Kwon, Hojeong Lee, Youngkook Kwon,* Sang Kyu Kwak,* and Jong-Beom Baek*

**Table of Contents**

Supplementary Figure S1-S36

Supplementary Table S1-S5

Supplementary References

**Figure S1.** Photograph of the as-synthesized catalyst placed on an analytical balance to verify the sample mass.

**Figure S2.** Schematic illustrations of the catalyst structures. (a) Ru_NC_@Fe_1_NC and (b) Ru_NC_@Ru_1_NC.

**Figure S3.** HAADF-STEM image of Ru_NC_@Ru_1_NC.

**Figure S4.** HAADF-STEM and corresponding elemental EDS mapping images of Ru_NC_@Fe_1_NC. (a) HAADF-STEM image. Elemental maps for (b) Ru, (c) Fe, (d) N, and (e) C.

**Figure S5.** HAADF-STEM and corresponding elemental EDS mapping images of Ru_NC_@Ru_1_NC. (a) HAADF-STEM image. Elemental maps for (b) Ru, (c) N, and (d) C.

**Figure S6.** Mössbauer spectrum of the as-prepared sample before thermal annealing.

**Figure S7.** XRD patterns of Ru_NC_@Fe_1_NC and Ru_NC_@Ru_1_NC.

**Figure S8.** (a) N 1s and (b) Ru 3p XPS spectra of Ru_NC_@Fe_1_NC.

**Figure S9.** (a) N 1s and (b) Ru 3p XPS spectra of Ru_NC_@Ru_1_NC.

**Figure S10.** k-Space X-ray absorption fine structure spectra at the Fe K-edge. (a) Ru_NC_@Fe_1_NC, (b) Fe_2_O_3_, and (c) Fe foil.

**Figure S11.** Wavelet transform analysis of the Fe K-edge EXAFS spectra. (a) Ru_NC_@Fe_1_NC, (b) Fe_2_O_3_, and (c) Fe foil.

**Figure S12.** Ru K-edge EXAFS spectra in k-space. (a) Ru foil, (b) RuO_2_, (c) Ru_NC_@Ru_1_NC, and (d) Ru_NC_@Fe_1_NC.

**Figure S13.** Wavelet transform analysis of Ru K-edge EXAFS spectra. (a) Ru_NC_@Fe_1_NC, (b) Ru_NC_@Ru_1_NC, (c) RuO_2_, and (d) Ru foil.

**Figure S14.** HER polarization curves of three specimens randomly sampled from different regions of a single ~10 g batch of Ru_NC_@Fe_1_NC in 1.0 m aqueous KOH.

**Figure S15.** HER polarization curves of Ru_NC_@Fe_1_NC, Ru_NC_@Ru_1_NC, Ru/C, and Fe_1_NC in 1.0 m aqueous KOH.

**Figure S16.** Cyclic voltammetry curves recorded at different scan rates for the evaluation of double-layer capacitance. (a) Pt/C, (b) Ru/C, (c) Ru_NC_@Ru_1_NC, and (d) Ru_NC_@Fe_1_NC.

**Figure S17.** Double-layer capacitances derived from CV curves.

**Figure S18.** ECSA-normalized polarization curves of Ru_NC_@Fe_1_NC, Ru_NC_@Ru_1_NC, commercial Ru/C, and Pt/C in 1.0 m aqueous KOH.

**Figure S19.** Mass activities of Ru_NC_@Fe_1_NC, Ru_NC_@Ru_1_NC, Ru/C, and Pt/C at overpotentials of 50 and 100 mV in 1.0 m aqueous KOH, measured under identical low-loading conditions.

**Figure S20.** Linear sweep voltammetry curves obtained during EDTA poisoning tests in alkaline electrolyte. (a) Pt/C, (b) Ru/C, (c) Ru_NC_@Ru_1_NC, and (d) Ru_NC_@Fe_1_NC. Solid lines denote the initial HER activity before EDTA addition, whereas dashed lines indicate the activity after EDTA introduction, highlighting the contribution of atomically dispersed metal sites.

**Figure S21.** Linear sweep voltammetry curves recorded for KSCN poisoning tests in alkaline electrolyte. (a) Pt/C, (b) Ru/C, (c) Ru_NC_@Ru_1_NC, and (d) Ru_NC_@Fe_1_NC. Solid lines correspond to measurements obtained before KSCN addition, while dashed lines represent the LSV curves recorded after the introduction of KSCN into the electrolyte.

**Figure S22.** Durability evaluation of Ru_NC_@Fe_1_NC toward the alkaline hydrogen evolution reaction. (a) Chronopotentiometry measurement conducted at a constant current density to assess the long-term operational stability of Ru_NC_@Fe_1_NC. (b) Linear sweep voltammetry curves of Ru_NC_@Fe_1_NC recorded before (solid line) and after (dashed line) 1000 continuous cyclic voltammetry cycles, demonstrating the electrochemical durability of the catalyst.

**Figure S23**. Photographs of (a) pristine nickel foam, (b) the Ru_NC_@Fe_1_NC-coated electrode prepared by electrospray deposition, and (c) the electrode after the 1,100 h AEMWE stability test.

**Figure S24**. (a–c) HAADF-STEM images of Ru_NC_@Fe_1_NC after the 1,100 h AEMWE stability test, acquired from different regions of the sample. The yellow circles indicate atomically dispersed Fe single atoms, and the Ru nanoclusters retain their ultrasmall size without noticeable aggregation. Scale bars: 2 nm.

**Figure S25.** HAADF-STEM and corresponding EDS elemental mapping images of Ru_NC_@Fe_1_NC after the 1,100 h AEMWE stability test. (a) HAADF-STEM image. Elemental maps for (b) C, (c) N, (d) Ru, (e) Ni, and (f) Fe. The Ni signal originates from the nickel foam substrate. Scale bar: 25 nm.

**Figure S26.** Comparison of the cell voltages at 1.0 A cm⁻^2^ of Ru_NC_@Fe_1_NC and recently reported AEMWE electrocatalysts. Detailed operating conditions and references are summarized in **Table S4**.

**Figure S27.** CO stripping cyclic voltammetry curves recorded in alkaline electrolyte. (a) Ru/C, (b) Ru_NC_@Ru_1_NC, and (c) Ru_NC_@Fe_1_NC. Light-colored curves correspond to background CVs recorded in the absence of adsorbed CO, whereas dark-colored curves represent CO stripping CVs obtained after CO adsorption, reflecting the oxidation of adsorbed CO species on the catalyst surfaces.

**Figure S28.** Anodic stripping voltammograms of Ru_NC_@Fe_1_NC, Ru_NC_@Ru_1_NC, and Fe_1_NC recorded in Ar-saturated 1.0 m aqueous KOH after hydrogen accumulation for 60 s at a mild cathodic potential.

**Figure S29.** Anodic stripping voltammograms recorded at various scan rates (10–200 mV s⁻^1^) after a fixed hydrogen accumulation time of 60 s. (a) Ru_NC_@Fe_1_NC, (b) Ru_NC_@Ru_1_NC, and (c) Fe_1_NC.

**Figure S30.** Desorption charge (Q), obtained by integrating the baseline-corrected anodic stripping curves of Ru_NC_@Fe_1_NC, as a function of scan rate (10–200 mV s⁻^1^). The dashed line indicates the average value (17.3 ± 0.8 mC cm⁻^2^).

**Figure S31.** Optimized structural models of the Ru_NC_@Fe_1_NC (left) and Ru_NC_@Ru_1_NC (right) catalysts considered for alkaline hydrogen evolution reaction (HER) calculation.

**Figure S32.** Calculated *OH adsorption energies on single atom sites in Ru_NC_@Fe_1_NC and Ru_NC_@Ru_1_NC. Note that Fe_1_ and Ru_1_ denote the Fe single-atom site and Ru single-atom site, respectively.

**Figure S33.** Mulliken charge analysis of the optimized structures for *H_2_O and *OH adsorbed on the Fe_1_ and Ru_1_ single-atom sites.

**Figure S34.** Schematic diagram of the synergistic effect mechanism among Fe_1_ site, and Ru_NC_ sites in Ru_NC_@Fe_1_NC systems in the alkaline HER process.

**Figure S35.** Calculated doping formation energies (*i.e.,* Δ*E*_Df_) of various N-doping configurations around the single atom site in Ru_NC_@Fe_1_NC (left) and Ru_NC_@Ru_1_NC (right).

**Figure S36.** Calculated binding energies (*i.e.,* Δ*E*_Bind_) between the Ru nanocluster and the single-atom-doped graphene for different configurations in Ru_NC_@Fe_1_NC and Ru_NC_@Ru_1_NC.

Table S1. C and N contents of the NC supports prepared at different ball-milling speeds, determined by elemental analysis.

| **Ball-milling speed (rpm)** | **C (wt%)** | **N (wt%)** |
| --- | --- | --- |
| 100 | 96.45 | N.D. |
| 200 | 93.33 | 3.04 |
| 300 (NC) | 90.51 | 7.05 |
| 500 (Fe_1_NC) | 86.98 | 7.47 |

* N.D.: not detected / The supports prepared at 300 and 500 rpm were used for Ru_NC_@Ru_1_NC and Ru_NC_@Fe_1_NC, respectively.

Table S2. ICP-OES results of the composition of Ru and Fe in the catalysts.

| **Sample** | **Ru (wt%)** | **Fe (wt%)** |
| --- | --- | --- |
| Ru_NC_@Fe_1_NC | 2.10 | 0.33 |
| Ru_NC_@Ru_1_NC | 1.99 | N.D. |

* N.D.: not detected

**Table S3.** EXAFS fitting parameters^a^ for the Fe K-edge of Ru_NC_@Fe_1_NC and the reference samples.

| **Sample** | **Path** | **N^b^** | **R (Å)^c^** | **σ^2^×10^3^ (Å^2^)^d^** | **ΔE_0_ (eV)^e^** | **R factor** |
| --- | --- | --- | --- | --- | --- | --- |
| Fe foil | Fe-Fe | 8 | 2.48±0.01 | 5.3±0.2 | 7.2±1.1 | 0.002 |
|  | Fe-Fe | 6 | 2.86±0.01 | 6.9±0.5 | 7.2±1.1 | 0.002 |
| Fe_2_O_3_ | Fe-O | 3 | 1.90±0.03 | 9.1±1.9 | −8.1±5.1 | 0.027 |
|  | Fe-O | 3 | 1.97±0.03 | 9.1±1.9 | −8.1±5.1 | 0.027 |
| Ru_NC_@Fe_1_NC | Fe-N | 3.7±1.1 | 2.00±0.04 | 6.0 | −4.8±8.3 | 0.049 |

^a^ S_o_^2^ was fixed at 0.8. ^b^ *N*: coordination numbers; ^c^ *R*: bond distance; ^d^ *σ^2^*: Debye-Waller factors; ^e^ *ΔE_0_*: the inner potential correction. R factor: goodness of fit according to the experimental EXAFS fit.

Table S4. Comparison of AEMWE performance, operating conditions, and long-term stability between the Ru_NC_@Fe_1_NC catalyst and other recently reported state-of-the-art electrocatalysts.

| Catalyst | | Cell voltage at 1 A cm^−2^ (V) | | Membrane type | | Temperature (°C) | Electrolyte | | | Stability | Ref | | |
| --- | --- | --- | --- | --- | --- | --- | --- | --- | --- | --- | --- | --- | --- |
| Ru_NC_@Fe_1_NC | 1.66 | | Sustainion X37-50 | | 80 | | | 1.0 m KOH | 1100 h @ 1A cm^−2^ | | | This work |  |
| HTS-Ru-NCs/TiN | 1.67 | | FAA-3-50 | | 60 | | | 1.0 m KOH | 170 h @ 1A cm^−2^ | | | [1] |  |
| GCB-NiFe | 1.68 | | Sustainion X37-50 | | 60 | | | 1.0 m KOH | 1000 h @ 0.5 A cm^−2^ | | | [2] |  |
| NiFeP | 1.68 | | KMem AM01-50 | | 60 | | | 1.0 m KOH | 120 h @ 0.5 A cm^−2^ | | | [3] |  |
| Li_3.0_RuSn_0.8_NWs | 1.689 | | PiperION A | | 80 | | | 1.0 m KOH | 1000 h @ 1A cm^−2^ | | | [4] |  |
| Co_2_FeW_1.6_O_x_ | 1.69 | | AEM, Grade T | | 80 | | | 1.0 m KOH | 600 h @ 1A cm^−2^ | | | [5] |  |
| RT-NiFe-LDH | 1.69 | | AF3-HWK9-75 | | 70 | | | 1.0 m KOH | 100 h @ 1A cm^−2^ | | | [6] |  |
| WS_2_ superstructure | 1.70 | | Sustainion X37-50 | | 60 | | | 30 wt% KOH | 1001 h @ 1A cm^−2^ | | | [7] |  |
| ac-Ce_SA_RuO_x_ | 1.71 | | Sustainion X37-50 | | 80 | | | 1.0 m KOH | 300 h @ 1A cm^−2^ | | | [8] |  |
| Pt_1_Co_6_/np-Co_2_P | 1.71 | | FAA-3-50 | | 80 | | | 1.0 m KOH | 150 h @ 1A cm^−2^ | | | [9] |  |
| Ni-BDC:Fc_5:1_ | 1.73 | | Sustainion X37–50 | | 60 | | | 1.0 m KOH | 120 h @ 1A cm^−2^ | | | [10] |  |
| NiFe_2_O_4_ | 1.73 | | AEM, Grade T | | 60 | | | 1.0 m KOH | 500 h @ 0.5 A cm^−2^ | | | [11] |  |
| Pt-HEA-cluster/CeO_2_/C | 1.74 | | KMem AM01 | | 80 | | | 1.0 m KOH | 500 h @ 0.5 A cm^−2^ | | | [12] |  |
| Ru–Ga_SA_/N–C | 1.74 | | Sustainion X37–50 | | 60 | | | 1.0 m KOH | 170 h @ 1A cm^−2^ | | | [13] |  |
| FeZnNNiCoV | 1.76 | | FAA-3-PK-130 | | 80 | | | 1.0 m KOH | 50 h @ 1A cm^−2^ | | | [14] |  |
| a-RNMO | 1.78 | | KMem AEM02-80 | | 80 | | | 1.0 m KOH | 100 h @ 1A cm^−2^ | | | [15] |  |
| N-CoO | 1.78 | | piperION A15R | | 60 | | | 1.0 m KOH | 300 h @ 1A cm^−2^ | | | [16] |  |
| MoNi_4_ \|\| Fe-Ni_2_P/NiMoO_4_ | 1.78 | | PiperION-A | | 20 | | | 1.0 m KOH | 100 h @ 1A cm^−2^ | | | [17] |  |
| (Ni,Fe)S_2_@Ti_3_C_2_ | 1.79 | | FAA-3-PK-130 | | 70 | | | 1.0 m KOH | 500 h @ 0.5 A cm^−2^ | | | [18] |  |
| Bi/BiCeO_1.8_H | 1.79 | | Sustainion X37-50 | | 50 | | | 1.0 m KOH | 100 h @ 1A cm^−2^ | | | [19] |  |
| d-NiOOH | 1.79 | | MTCP-50 | | 80 | | | 1.0 m KOH | 100 h @ 1A cm^−2^ | | | [20] |  |
| JH-Pt_2_Tb/C | 1.79 | | Sustainion X37-50 | | 80 | | | 1.0 m KOH | 100 h @ 1A cm^−2^ | | | [21] |  |
| NiFeOOH | 1.79 | | Versogen | | 60 | | | 1.0 m KOH | 150 h @ 1A cm^−2^ | | | [22] |  |
| Ni_x_Fe_1−x_S_2_ | 1.79 | | AF1-HNN8-50 | | 60 | | | 1.0 m KOH | 550 h @ 1A cm^−2^ | | | [23] |  |
| Ru SAs/WC_x_ | 1.79 | | Sustainion X37-50 | | 80 | | | 1.0 m KOH | 190 h @ 1A cm^−2^ | | | [24] |  |
| Ru-Ni_3_N | 1.79 | | FAA-3-50 | | 60 | | | 1.0 m KOH | 550 h @ 0.5 A cm^−2^ | | | [25] |  |
| Pt(OH)(O_3_)/Co(P) | 1.80 | | Alkymer | | 80 | | | 1.0 m KOH | 550 h @ 1.9 V | | | [26] |  |
| Li_2_Mn_1_−xRu_x_O_3_ | 1.82 | | AF2-HLF8-25X | | 60 | | | 0.1 m KOH | - | | | [27] |  |
| Pt_5.3%_-Ru_fcc_ | 1.82 | | Sustainion X37-50 | | 80 | | | 1.0 m KOH | 400 h @ 1A cm^−2^ | | | [28] |  |
| LFA | 1.83 | | FAA-3-50 | | 50 | | | 1.0 m KOH | 1300 h @ 2A cm^−2^ | | | [29] |  |
| CoN/VN@NF | 1.84 | | FAA-3-PK-130 | | 70 | | | 1.0 m KOH | 1000 h @ 0.5 A cm^−2^ | | | [30] |  |
| NiFe-SQ/NF-R | 1.85 | | FAA-3-50 | | 25 | | | 1.0 m KOH | 100 h @ 1A cm^−2^ | | | [31] |  |
| Pt-Ru/RuO_2_ | 1.90 | | AEMION^TM^ | | 60 | | | 1.0 m KOH | - | | | [32] |  |
| CdNNi_3_ | 1.90 | | MTCP-50 | | 60 | | | 1.0 m KOH | 100 h @ 0.5 A cm^−2^ | | | [33] |  |
| MgO_x_/MoO_y_-Ru | 1.90 | | FAA-3-20 | | - | | | 1.0 m KOH | 60 h @ 0.5 A cm^−2^ | | | [34] |  |
| Pt-AC/Cr–N–C | 1.90 | | Sustainion X37-50 | | 80 | | | 1.0 m KOH | 100 h @ 0.5 A cm^−2^ | | | [35] |  |
| Ru_1_/PNC | 1.94 | | FAA-3-50 | | 60 | | | 1.0 m KOH | 266 h @ 2A cm^−2^ | | | [36] |  |
| CoCrO_x_ | 1.95 | | PiperION-A60-HCO3 | | 60 | | | 1.0 m KOH | 120 h @ 0.5 A cm^−2^ | | | [37] |  |
| Pt-MoAl_1−x_B | 2.00 | | Sustainion X37-50 | | 60 | | | 1.0 m KOH | 280 h @ 0.5 A cm^−2^ | | | [38] |  |
| W-Co_3_O_4_ | 2.12 | | Versogen | | 60 | | | 1.0 m KOH | 100 h @ 0.2 A cm^−2^ | | | [39] |  |
| Co_SA_Ru_AC_@NCB | 2.16 | | FAA-3-50 | | 55 | | | 1.0 m KOH | 1000 h @ 1 A cm^−2^ | | | [40] |  |
| *A*-Fe_2_S_1_N_5_/SNC | 2.18 | | Sustainion X37-50 | | 20 | | | 1.0 m KOH | 250 h @ 0.5 A cm^−2^ | | | [41] |  |
| Ru/Mo_2_C/MoO_2_ | 2.20 | | PiperION A80 | | 60 | | | 1.0 m KOH | 80 h @ 0.1 A cm^−2^ | | | [42] |  |
| Ir/HfO_2_@C | 2.22 | | FAA-3-PK-130 | | - | | | 1.0 m KOH | 22 h @ 0.25 A cm^−2^ | | | [43] |  |
| NiFe-LS | 2.30 | | Sustainion X37-50 | | 20 | | | 1.0 m KOH | 140 h @ 0.5 A cm^−2^ | | | [44] |  |
| HfO_x_-in-Ir SSC | 2.31 | | FSSC-3-PK-130 | | 20 | | | 1.0 m KOH | 50 h @ 1 A cm^−2^ | | | [45] |  |

Table S5. DFT-Raman frequencies for Fe_1_ (Ru_NC_@Fe_1_NC) and Ru_1_ (Ru_NC_@Ru_1_NC) catalysts under H_2_O-adsorbed single-atom states.

| Catalyst Sample | Calculated Raman Shifts under H_2_O Adsorption (cm^−1^) |
| --- | --- |
| Fe_1_ (Ru_NC_@Fe_1_NC) | **1467**, 1447, 1427, 1404, 1362, 1321 |
| Ru_1_ (Ru_NC_@Ru_1_NC) | 1485, 1418, 1372, 1339 |

**References**

[1]	Zong, L., Lu, F., Li, P., et al., "Thermal Shock Synthesis for Loading Sub-2 nm Ru Nanoclusters on Titanium Nitride as a Remarkable Electrocatalyst toward Hydrogen Evolution Reaction," *Advanced Materials* 36, no. 32 (2024), https://doi.org/https://doi.org/10.1002/adma.202403525.

[2]	Zhang, T., Lin, S.-a., Liu, H., et al., "An Ionomer-Free Gapless Catalyst-Bridging Membrane Electrode Assembly for High-Performance Pure Water-Fed Anion Exchange Membrane Electrolyzer," *Advanced Materials* 38, no. 4 (2026), https://doi.org/https://doi.org/10.1002/adma.202509805.

[3]	Qian, Z.-X., Liang, G.-H., Shen, L.-F., et al., "Phase Engineering Facilitates O–O Coupling via Lattice Oxygen Mechanism for Enhanced Oxygen Evolution on Nickel–Iron Phosphide," *Journal of the American Chemical Society* 147, no. 1 (2025), https://doi.org/10.1021/jacs.4c15847.

[4]	Mao, J., Liang, J., Li, Y., et al., "Electrochemical Lithiation Regulates the Active Hydrogen Supply on Ru–Sn Nanowires for Hydrogen Evolution Toward the High-Performing Anion Exchange Membrane Water Electrolyzer," *Journal of the American Chemical Society* 147, no. 9 (2025), https://doi.org/10.1021/jacs.4c17373.

[5]	Gao, G., Li, G., Huang, T., et al., "Overcoming the Activity-Stability Trade-Off in Electrocatalysts via Unconventional Two-Step Structural Reconstructions of Amorphous Oxides," *Journal of the American Chemical Society* 147, no. 33 (2025), https://doi.org/10.1021/jacs.5c04970.

[6]	Seijas-Da Silva, A., Hartert, A., Oestreicher, V., et al., "Scalable synthesis of NiFe-layered double hydroxide for efficient anion exchange membrane electrolysis," *Nature Communications* 16, no. 1 (2025), https://doi.org/10.1038/s41467-025-61356-2.

[7]	Xie, L., Wang, L., Liu, X., et al., "Flexible tungsten disulfide superstructure engineering for efficient alkaline hydrogen evolution in anion exchange membrane water electrolysers," *Nature Communications* 15, no. 1 (2024), https://doi.org/10.1038/s41467-024-50117-2.

[8]	Chen, X., Bi, M., Yan, Q., et al., "Ce Single Atom-Engineered Amorphous/Crystalline Nanosheets for Enhanced Alkaline Water Electrolysis," *Advanced Materials* 37, no. 50 (2025), https://doi.org/https://doi.org/10.1002/adma.202508893.

[9]	Jiang, K., Liu, Z., Wang, Z., Xie, F., Yuan, X., Tan, Y., "Manipulating Interfacial Water Via Metallic Pt1Co6 Sites on Self-Adaptive Metal Phosphides to Enhance Water Electrolysis," *Advanced Materials* 37, no. 18 (2025), https://doi.org/https://doi.org/10.1002/adma.202419644.

[10]	Xu, H., Zheng, D. J., Wang, S., et al., "Stable Metal–Organic Electrocatalysts for Anion-Exchange Membrane Water Electrolyzers by Defect Engineering," *Journal of the American Chemical Society* 147, no. 33 (2025), https://doi.org/10.1021/jacs.5c06156.

[11]	Yoon, K.-Y., Lee, K.-B., Jeong, J., et al., "Improved Oxygen Evolution Reaction Kinetics with Titanium Incorporated Nickel Ferrite for Efficient Anion Exchange Membrane Electrolysis," *ACS Catalysis* 14, no. 7 (2024), https://doi.org/10.1021/acscatal.3c05761.

[12]	Wang, H., Wang, X., Gao, F., et al., "Synergistic Catalysis of Pt-Based High-Entropy Clusters Coupled with Super-Hydrophilic CeO2 Enables Efficient Anion Exchange Membrane Water Electrolysis," *Advanced Materials* 38, no. 2 (2026), https://doi.org/https://doi.org/10.1002/adma.202514269.

[13]	Zhou, C., Shi, J., Dong, Z., et al., "Oxophilic gallium single atoms bridged ruthenium clusters for practical anion-exchange membrane electrolyzer," *Nature Communications* 15, no. 1 (2024), https://doi.org/10.1038/s41467-024-51200-4.

[14]	Zhang, J., Tu, Y., Xu, X., et al., "A High-entropy Antiperovskite Nitride Enables Efficient Anion Exchange Membrane Water Electrolysis," *Advanced Materials* 37, no. 38 (2025), https://doi.org/https://doi.org/10.1002/adma.202509042.

[15]	Li, J., Zhu, Y., Li, C., et al., "Boosting and stabilizing oxygen evolution reaction through Ru single atoms anchored amorphous NiMoOx electrocatalyst," *Nature Communications* 16, no. 1 (2025), https://doi.org/10.1038/s41467-025-63870-9.

[16]	Yu, P.-C., Zhang, X.-L., Zhang, T.-Y., et al., "Nitrogen-Mediated Promotion of Cobalt-Based Oxygen Evolution Catalyst for Practical Anion-Exchange Membrane Electrolysis," *Journal of the American Chemical Society* 146, no. 29 (2024), https://doi.org/10.1021/jacs.4c05983.

[17]	Tao, X., Hou, L., Wang, X., Jin, J., Li, H., Gao, F., "Iron and oxygen vacancies co-modulated adsorption evolution and lattice oxygen dual-path mechanism for water oxidation," *Nature Communications* 16, no. 1 (2025), https://doi.org/10.1038/s41467-025-63844-x.

[18]	Wang, J., Liu, Y., Yang, G., et al., "MXene-Assisted NiFe sulfides for high-performance anion exchange membrane seawater electrolysis," *Nature Communications* 16, no. 1 (2025), https://doi.org/10.1038/s41467-025-56639-7.

[19]	Jo, S., Jeon, J. I., Shin, K. H., et al., "Stabilization of Lattice Oxygen Evolution Reactions in Oxophilic Ce-Mediated Bi/BiCeO1.8H Electrocatalysts for Efficient Anion Exchange Membrane Water Electrolyzers," *Advanced Materials* 36, no. 27 (2024), https://doi.org/https://doi.org/10.1002/adma.202314211.

[20]	Qi, S., You, J., Liufu, X., et al., "Switching the Oxygen Evolution Reaction Mechanism through the Creation of Disordered NiOOH Induced by Electrochemical Reconstruction," *Advanced Materials* 38, no. 5 (2026), https://doi.org/https://doi.org/10.1002/adma.202512188.

[21]	Yan, W., Mou, Y., Li, M., et al., "C15-Phase Platinum-Lanthanide Intermetallics for Efficient Hydrogen Evolution: Identifying Lanthanide's Enhanced Mechanism," *Advanced Materials* 37, no. 34 (2025), https://doi.org/https://doi.org/10.1002/adma.202506936.

[22]	Wu, Z.-P., Zuo, S., Wu, R., et al., "Potential Pulsing-Induced Fe Undercoordination in NiFe (Oxy)Hydroxide Enhances Electrochemical Oxygen Evolution," *Journal of the American Chemical Society* 147, no. 40 (2025), https://doi.org/10.1021/jacs.5c12507.

[23]	Xia, L., Gomes, B. F., Jiang, W., et al., "Operando-informed precatalyst programming towards reliable high-current-density electrolysis," *Nature Materials* 24, no. 5 (2025), https://doi.org/10.1038/s41563-025-02128-7.

[24]	Lin, X., Hu, W., Xu, J., et al., "Alleviating OH Blockage on the Catalyst Surface by the Puncture Effect of Single-Atom Sites to Boost Alkaline Water Electrolysis," *Journal of the American Chemical Society* 146, no. 7 (2024), https://doi.org/10.1021/jacs.3c13676.

[25]	Qiu, L., Tian, F., He, L., et al., "Robust Interfacial Hydrogen-Bond Network on Positively Charged Ru-N-Ni Dual Sites Boosts Alkaline Hydrogen Electrocatalysis," *Advanced Materials* 38, no. 12 (2026), https://doi.org/https://doi.org/10.1002/adma.202512568.

[26]	Zeng, L., Zhao, Z., Lv, F., et al., "Anti-dissolution Pt single site with Pt(OH)(O3)/Co(P) coordination for efficient alkaline water splitting electrolyzer," *Nature Communications* 13, no. 1 (2022), https://doi.org/10.1038/s41467-022-31406-0.

[27]	Zhong, X., Sui, L., Yang, M., et al., "Stabilization of layered lithium-rich manganese oxide for anion exchange membrane fuel cells and water electrolysers," *Nature Catalysis* 7, no. 5 (2024), https://doi.org/10.1038/s41929-024-01136-1.

[28]	Jiang, W., Zhang, S. L., Yang, J., et al., "Phase-Regulatable Synthesis of Single-Atom Alloy Nanocages for Efficient Alkaline Hydrogen Evolution," *Journal of the American Chemical Society* 147, no. 39 (2025), https://doi.org/10.1021/jacs.5c06662.

[29]	Wang, J., Liang, C., Ma, X., et al., "Dynamically Adaptive Bubbling for Upgrading Oxygen Evolution Reaction Using Lamellar Fern-Like Alloy Aerogel Self-Standing Electrodes," *Advanced Materials* 36, no. 1 (2024), https://doi.org/https://doi.org/10.1002/adma.202307925.

[30]	Liang, Z., Shen, D., Wei, Y., et al., "Modulating the Electronic Structure of Cobalt-Vanadium Bimetal Catalysts for High-Stable Anion Exchange Membrane Water Electrolyzer," *Advanced Materials* 36, no. 41 (2024), https://doi.org/https://doi.org/10.1002/adma.202408634.

[31]	Fan, R., Lu, S., Wang, F., et al., "Enhancing catalytic durability in alkaline oxygen evolution reaction through squaric acid anion intercalation," *Nature Communications* 16, no. 1 (2025), https://doi.org/10.1038/s41467-025-58623-7.

[32]	Zhu, Y., Klingenhof, M., Gao, C., et al., "Facilitating alkaline hydrogen evolution reaction on the hetero-interfaced Ru/RuO2 through Pt single atoms doping," *Nature Communications* 15, no. 1 (2024), https://doi.org/10.1038/s41467-024-45654-9.

[33]	Chen, Y., Tang, Z., Liu, Z., et al., "Toward the Ideal Alkaline Hydrogen Evolution Electrocatalyst: a Noble Metal-Free Antiperovskite Optimized with A-Site Tuning," *Advanced Materials* 37, no. 29 (2025), https://doi.org/https://doi.org/10.1002/adma.202504607.

[34]	Tian, F., Geng, S., Li, M., et al., "Synergetic Oxidized Mg and Mo Sites on Amorphous Ru Metallene Boost Hydrogen Evolution Electrocatalysis," *Advanced Materials* 37, no. 18 (2025), https://doi.org/https://doi.org/10.1002/adma.202501230.

[35]	Zeng, L., Zhao, Z., Huang, Q., et al., "Single-Atom Cr–N4 Sites with High Oxophilicity Interfaced with Pt Atomic Clusters for Practical Alkaline Hydrogen Evolution Catalysis," *Journal of the American Chemical Society* 145, no. 39 (2023), https://doi.org/10.1021/jacs.3c06863.

[36]	Jin, H., Chen, X., Da, Y., et al., "Identifying the Bifunctional Mechanism in Alkaline Water Electrolysis by Lewis Pairs at the Single-Atom Scale," *Journal of the American Chemical Society* 147, no. 4 (2025), https://doi.org/10.1021/jacs.4c18040.

[37]	Li, S., Liu, T., Zhang, W., et al., "Highly efficient anion exchange membrane water electrolyzers via chromium-doped amorphous electrocatalysts," *Nature Communications* 15, no. 1 (2024), https://doi.org/10.1038/s41467-024-47736-0.

[38]	Park, S. J., Nguyen, T. H., Tran, D. T., Dinh, V. A., Lee, J. H., Kim, N. H., "Delaminated MBene sheets beyond usual 2D transition metal materials for securing Pt single atoms to boost hydrogen evolution," *Energy & Environmental Science* 16, no. 9 (2023), https://doi.org/10.1039/D3EE01314F.

[39]	Wang, Y., Jia, B., Qin, W., et al., "High-Density W Single Atoms in Two-Dimensional Spinel Oxide Break the Structural Integrity for Enhanced Oxygen Evolution Catalysis," *Journal of the American Chemical Society* 147, no. 35 (2025), https://doi.org/10.1021/jacs.5c12122.

[40]	Yang, X., Song, W., Liao, K., et al., "Cohesive energy discrepancy drives the fabrication of multimetallic atomically dispersed materials for hydrogen evolution reaction," *Nature Communications* 15, no. 1 (2024), https://doi.org/10.1038/s41467-024-52520-1.

[41]	Zhang, L., Zhang, N., Shang, H., et al., "High-density asymmetric iron dual-atom sites for efficient and stable electrochemical water oxidation," *Nature Communications* 15, no. 1 (2024), https://doi.org/10.1038/s41467-024-53871-5.

[42]	Hou, L., Li, C., Jang, H., et al., "Rationally Designed Mo/Ru-Based Multi-Site Heterogeneous Electrocatalyst for Accelerated Alkaline Hydrogen Evolution Reaction," *Advanced Materials* 36, no. 48 (2024), https://doi.org/https://doi.org/10.1002/adma.202410039.

[43]	Shao, W., Xing, Z., Xu, X., et al., "Bioinspired Proton Pump on Ferroelectric HfO2-Coupled Ir Catalysts with Bidirectional Hydrogen Spillover for pH-Universal and Superior Hydrogen Production," *Journal of the American Chemical Society* 146, no. 40 (2024), https://doi.org/10.1021/jacs.4c08100.

[44]	Wang, Y., Li, S., Hou, X., et al., "Low-Spin Fe3+ Evoked by Multiple Defects with Optimal Intermediate Adsorption Attaining Unparalleled Performance in Water Oxidation," *Advanced Materials* 36, no. 52 (2024), https://doi.org/https://doi.org/10.1002/adma.202412598.

[45]	Shao, W., Xing, Z., Zhou, M., et al., "Local Proton-Rich Solid Solution Catalyst with a Cluster-in-Cluster Structure for Anti-interference Seawater Splitting," *Advanced Materials* 37, no. 38 (2025), https://doi.org/https://doi.org/10.1002/adma.202507080.
